# Supplementary material for: Engineering compact bacterial combinatorial promoters for two-input chemical AND switching
Source: J Biol Eng. 2026 Jul 27;20:153. doi: 10.1186/s13036-026-00728-8 (PMC13688774; doi:10.1186/s13036-026-00728-8)
Supplement: Supplementary file 1 [file 13036_2026_728_MOESM4_ESM.pdf]

Supplementary Information: Engineering compact bacterial combinatorial promoters for two-input chemical AND switching

Satya Prakash[1,2] and Alfonso Jaramillo[1,3†]

[1] Jaramillo Lab. Warwick Integrative Synthetic Biology Centre and School of Life Sciences, University of Warwick, Coventry CV4 7AL, UK.

[2] Department of Life Sciences, Imperial College London, London SW7 2AZ, UK.

[3] Jaramillo Lab, i2SysBio, CSIC-University of Valencia, Paterna, Spain. Alfonso.Jaramillo@csic.es.

[†] Corresponding author and lead contact: Prof. Alfonso Jaramillo. *De novo* Synthetic Biology Lab (Jaramillo Lab), i2SysBio, CSIC-University of Valencia, Paterna, Spain. Alfonso.Jaramillo@csic.es.

SUPPLEMENTARY TEXT 3
SUPPLEMENTARY FIGURES 4
SUPPLEMENTARY TABLES 24
REFERENCES 44

Supplementary Figures

Supplementary Fig. S1 | Dynamics and fit diagnostics for pTet-PhlF.
Supplementary Fig. S2 | Dynamics and fit diagnostics for pBet-Tac.
Supplementary Fig. S3 | Dynamics and fit diagnostics for pBet-Van.
Supplementary Fig. S4 | Dynamics and fit diagnostics for pPhlF-Tac.
Supplementary Fig. S5 | Dynamics and fit diagnostics for pPhlF-Tet.
Supplementary Fig. S6 | Dynamics and fit diagnostics for pTac-Tet.
Supplementary Fig. S7 | Dynamics and fit diagnostics for pTet-Bet.
Supplementary Fig. S8 | Dynamics and fit diagnostics for pTet-Ttg.
Supplementary Fig. S9 | Dynamics and fit diagnostics for pTet-Van.
Supplementary Fig. S10 | Dynamics and fit diagnostics for pTtg-Tac.
Supplementary Fig. S11 | Dynamics and fit diagnostics for pVan-Tac.
Supplementary Fig. S12 | Dynamics and fit diagnostics for pVan-Ttg.
Supplementary Fig. S13 | Reporter-specific truth tables matching Supplementary Table S2.
Supplementary Fig. S14 | Reporter-specific truth tables matching Supplementary Table S2.
Supplementary Fig. S15 | Reporter-specific truth tables matching Supplementary Table S2.
Supplementary Fig. S16 | Reporter-specific truth tables matching Supplementary Table S2.
Supplementary Fig. S17 | Architecture of the experimentally tested pTac-Van combinatorial promoter.
Supplementary Fig. S18 | Coverage of the scaffold-by-input design space used in this study.
Supplementary Fig. S19 | Sequence alignment around the -10 region and +1 site in reverse-operator examples.

Supplementary Tables

Supplementary Table S1 | Distributed source-data and reproducibility files.
Supplementary Table S2 | Statistics for all 19 curated two-input combinatorial promoter-reporter truth tables, including the Engineering Utility flag.
Supplementary Table S3 | Excluded entries and quality-control summary for the primary analysis.
Supplementary Table S4 | Primers used in this work.
Supplementary Table S5 | Promoter sequences.
Supplementary Table S6 | Selected inducer concentrations and truth-table digit mapping.
Supplementary Table S7 | Compact promoter sequence annotations for engineered promoter architectures.
Supplementary Table S8 | Cross-reference between characterised promoter/reporter constructs.
Supplementary Table S9 | Representative mCherry plasmid sequence.
Supplementary Table S10 | Representative EGFP plasmid sequence.
Supplementary Table S11 | Overview of the engineered promoter architectures.
Supplementary Table S12 | Template-promoter YES-gate activation ratios and AND-promoter conditional activation ratios.
Supplementary Table S13 | Same-channel alternate-reporter background diagnostic and margin checks.

Supplementary Text

This Supplementary Information documents the inducer-conditioned truth-table analysis of the Tecan plate-reader datasets used to evaluate the engineered two-input combinatorial promoters. The response variable is the fitted normalised fluorescence (a.u./OD), extracted separately for GFP and RFP reporters. As in the main text, truth-table language is operational in inducer space: the experimentally controlled inputs are inducer concentrations, whereas promoter regulation in the stricter biophysical sense depends on intracellular concentrations of active transcription factors and promoter occupancy. We restrict the curated comparative analysis to the 12 pre-specified architectures that yielded complete four-state truth tables, while also documenting the experimentally tested exploratory pTac-Van construct separately. Supplementary Figures S1-S12 provide compact dynamics and fit diagnostics, Supplementary Figures S13-S19 provide reporter-specific truth tables, exploratory architecture, coverage, and sequence-context views, and Supplementary Tables S1-S13 provide replicate provenance, summary statistics, annotated promoter architectures, primer sequences, representative plasmid sequences, and construct-to-figure mapping. Promoter names follow the nomenclature used in the main text: engineered promoters are named by scaffold followed by the added input, whereas template promoters retain their original designations (pVanCC, pBetI, pTtg, pPhlF, pTac, and pTet*)13.

Dynamics and fit diagnostics for curated combinatorial designs. Only the 12 curated combinatorial promoters selected for truth-table evaluation are shown here. Each promoter is condensed to a single page with OD_{600}, GFP, and RFP trajectories overlaid by inducer state ([00], [10], [01], [11]), the fitted growth model overlaid on OD_{600}, and a fluorescence-versus-OD diagnostic panel for the reporter used in the promoter call. Fluorescence normalisation is assessed against measured OD_{600} within the OD window 0.15-0.70. Single-input template controls and exploratory constructs are summarised separately in the revised main-text figures and supplementary overview panels.

Supplementary Figures

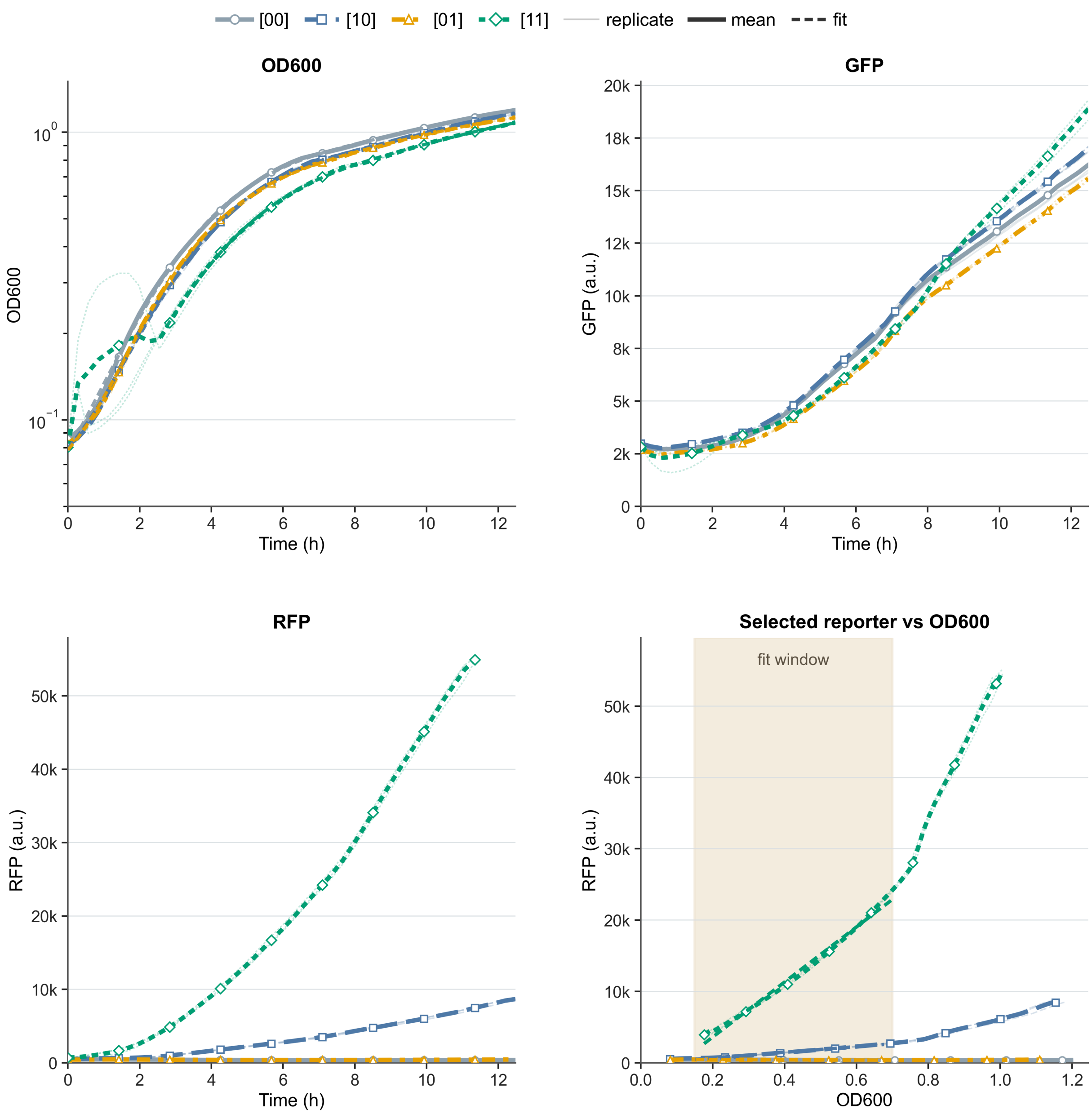

Supplementary Fig. S1 | Dynamics and fit diagnostics for pTet-PhlF. The four subpanels show OD_{600} versus time with the growth fit, GFP versus time, RFP versus time, and selected-reporter fluorescence versus OD_{600}. Selected reporter: RFP. Inducer pair, using the source grid label with units in Supplementary Table S6: aTc=0.2; DAPG=10. Biological replicates per state: [00] n = 3, [10] n = 3, [01] n = 3, [11] n = 3. GFP and RFP are independent reporter outputs, not normalisation controls. The fluorescence-versus-OD diagnostic is the panel used to derive the normalised fluorescence for the selected reporter.

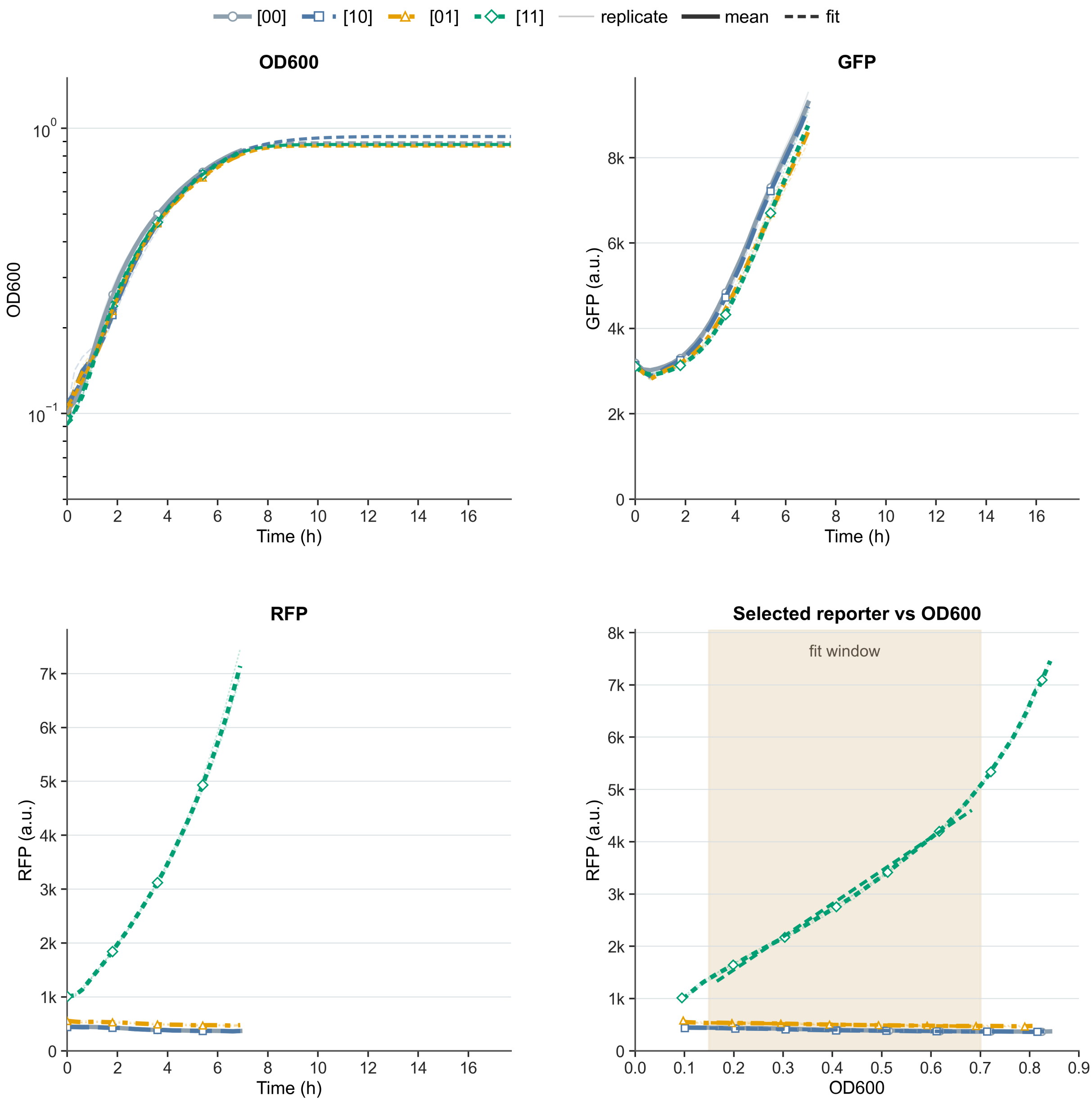

Supplementary Fig. S2 | Dynamics and fit diagnostics for pBet-Tac. The four subpanels show OD_{600} versus time with the growth fit, GFP versus time, RFP versus time, and selected-reporter fluorescence versus OD_{600}. Selected reporter: RFP. Inducer pair, using the source grid label with units in Supplementary Table S6: Cho=10000; IPTG=500. Biological replicates per state: [00] n = 3, [10] n = 3, [01] n = 3, [11] n = 3. GFP and RFP are independent reporter outputs, not normalisation controls. The fluorescence-versus-OD diagnostic is the panel used to derive the normalised fluorescence for the selected reporter.

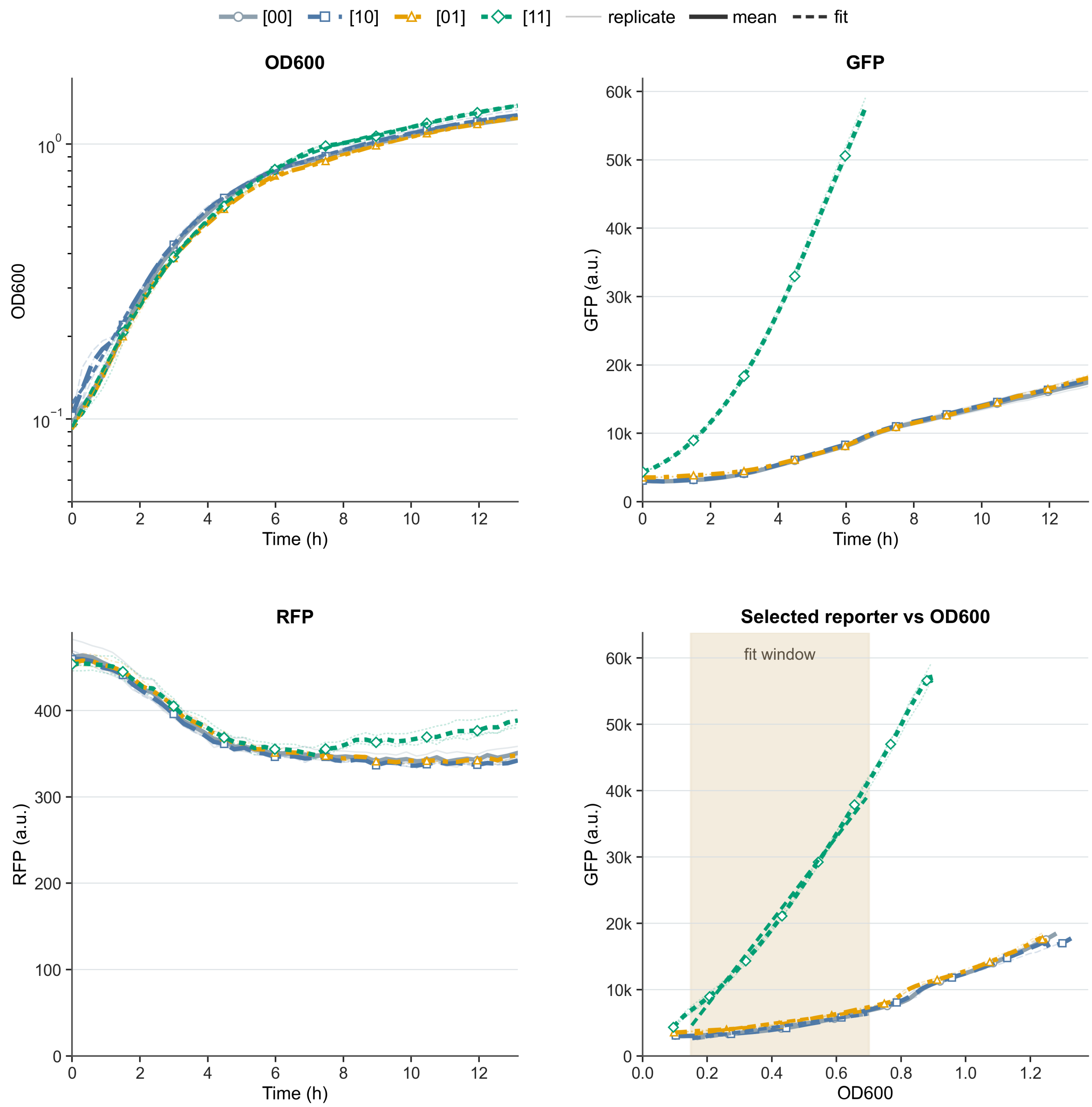

Supplementary Fig. S3 | Dynamics and fit diagnostics for pBet-Van. The four subpanels show OD_{600} versus time with the growth fit, GFP versus time, RFP versus time, and selected-reporter fluorescence versus OD_{600}. Selected reporter: GFP. Inducer pair, using the source grid label with units in Supplementary Table S6: Cho=10000; Van=100. Biological replicates per state: [00] n = 3, [10] n = 3, [01] n = 3, [11] n = 3. GFP and RFP are independent reporter outputs, not normalisation controls. The fluorescence-versus-OD diagnostic is the panel used to derive the normalised fluorescence for the selected reporter.

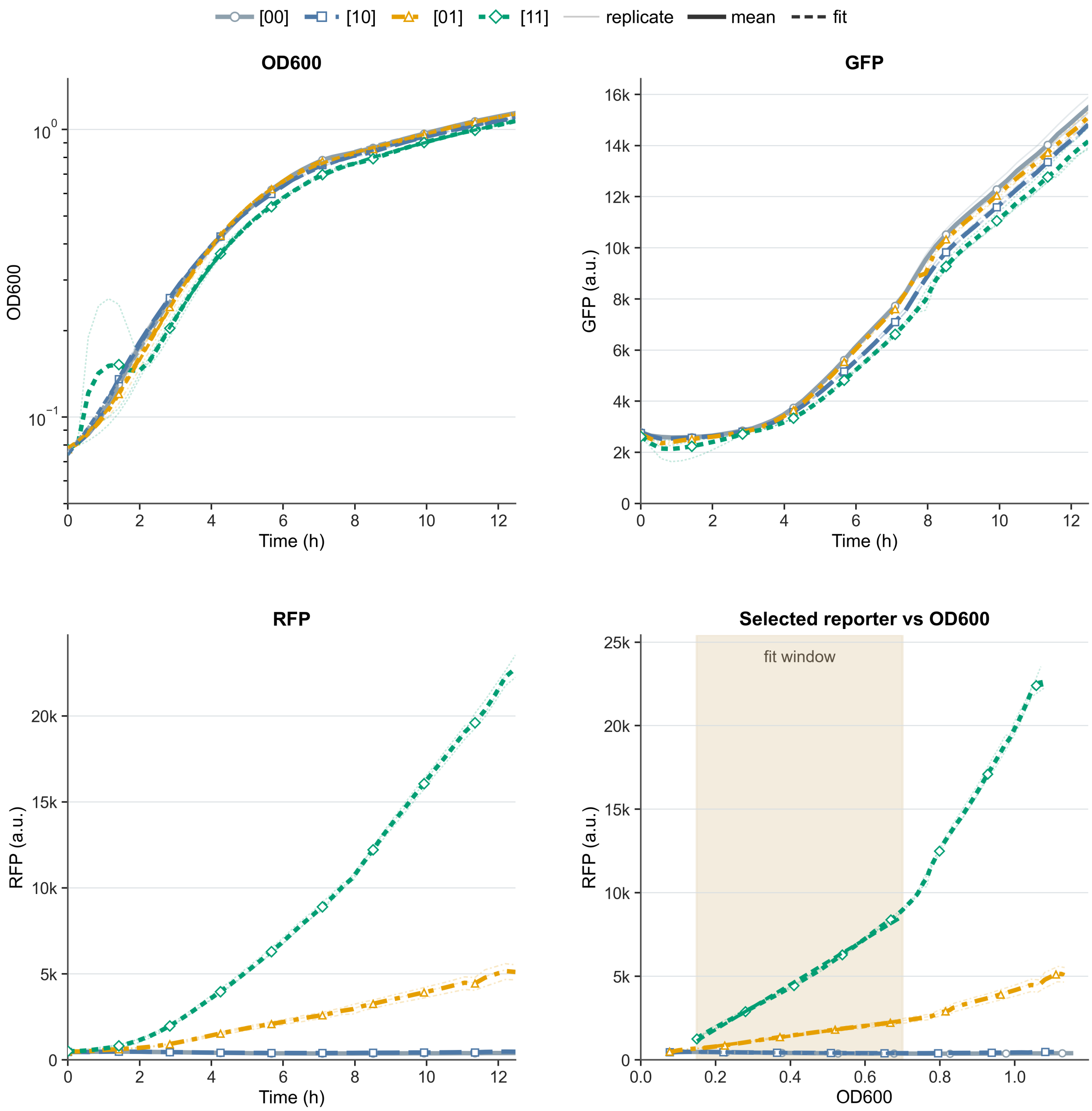

Supplementary Fig. S4 | Dynamics and fit diagnostics for pPhlF-Tac. The four subpanels show OD_{600} versus time with the growth fit, GFP versus time, RFP versus time, and selected-reporter fluorescence versus OD_{600}. Selected reporter: RFP. Inducer pair, using the source grid label with units in Supplementary Table S6: DAPG=10; IPTG=1000. Biological replicates per state: [00] n = 3, [10] n = 3, [01] n = 3, [11] n = 3. GFP and RFP are independent reporter outputs, not normalisation controls. The fluorescence-versus-OD diagnostic is the panel used to derive the normalised fluorescence for the selected reporter.

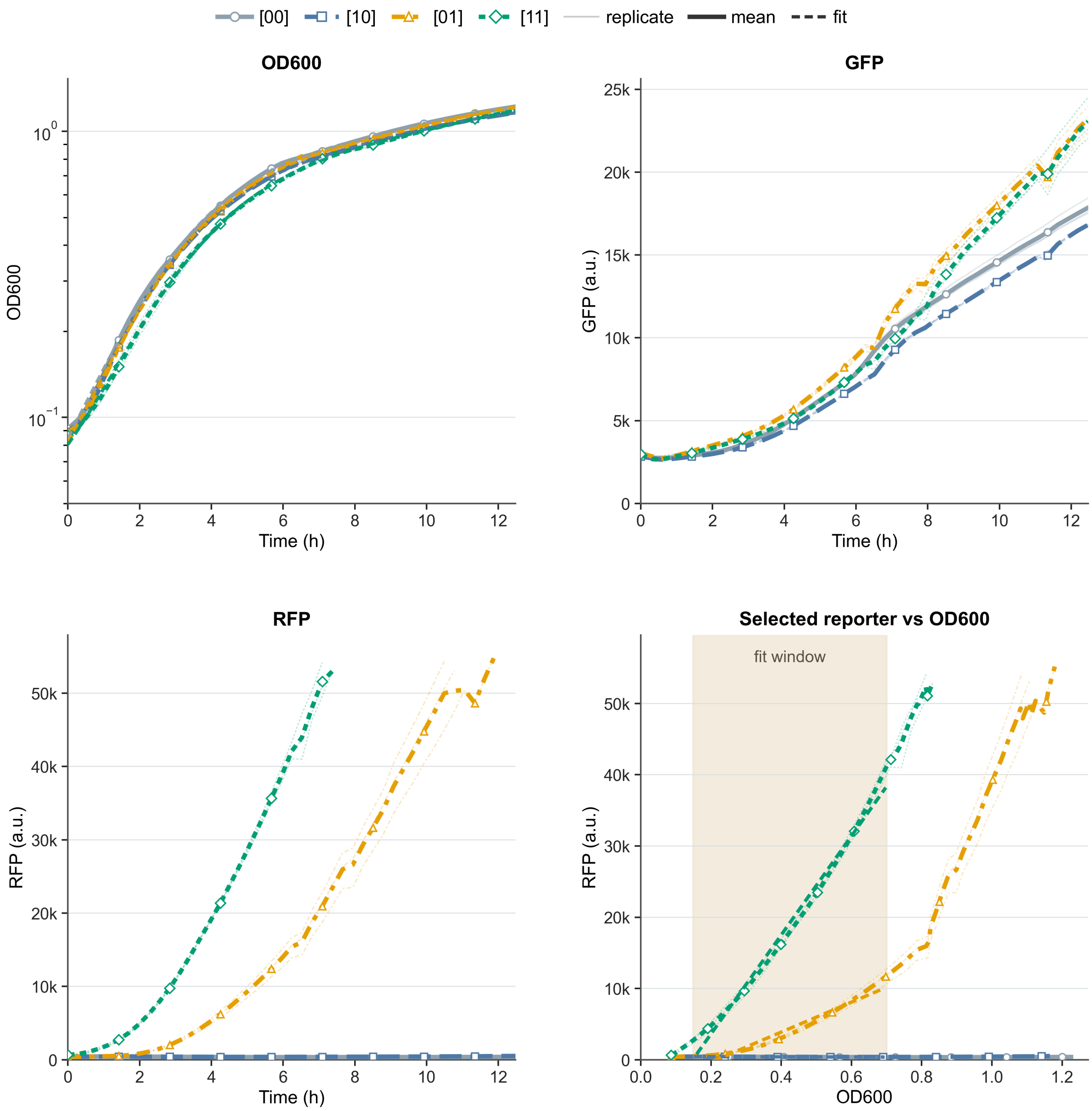

Supplementary Fig. S5 | Dynamics and fit diagnostics for pPhlF-Tet. The four subpanels show OD_{600} versus time with the growth fit, GFP versus time, RFP versus time, and selected-reporter fluorescence versus OD_{600}. Selected reporter: RFP. Inducer pair, using the source grid label with units in Supplementary Table S6: DAPG=10; aTc=0.2. Biological replicates per state: [00] n = 3, [10] n = 3, [01] n = 3, [11] n = 3. GFP and RFP are independent reporter outputs, not normalisation controls. The fluorescence-versus-OD diagnostic is the panel used to derive the normalised fluorescence for the selected reporter.

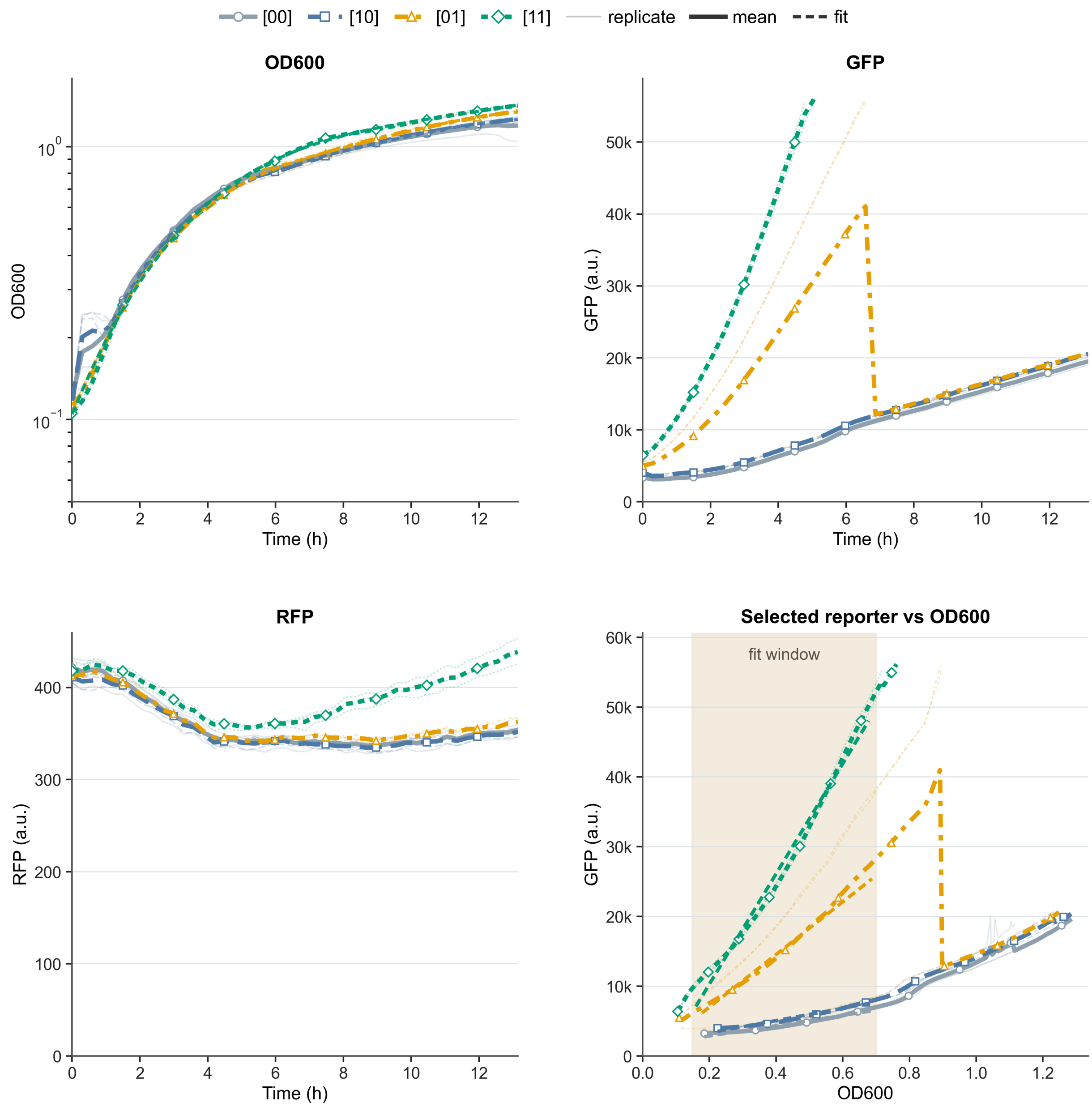

Supplementary Fig. S6 | Dynamics and fit diagnostics for pTac-Tet. The four subpanels show OD_{600} versus time with the growth fit, GFP versus time, RFP versus time, and selected-reporter fluorescence versus OD_{600}. Selected reporter: GFP. Inducer pair, using the source grid label with units in Supplementary Table S6: IPTG=1000; aTc=0.2. Biological replicates per state: [00] n = 3, [10] n = 3, [01] n = 3, [11] n = 3. GFP and RFP are independent reporter outputs, not normalisation controls. The fluorescence-versus-OD diagnostic is the panel used to derive the normalised fluorescence for the selected reporter.

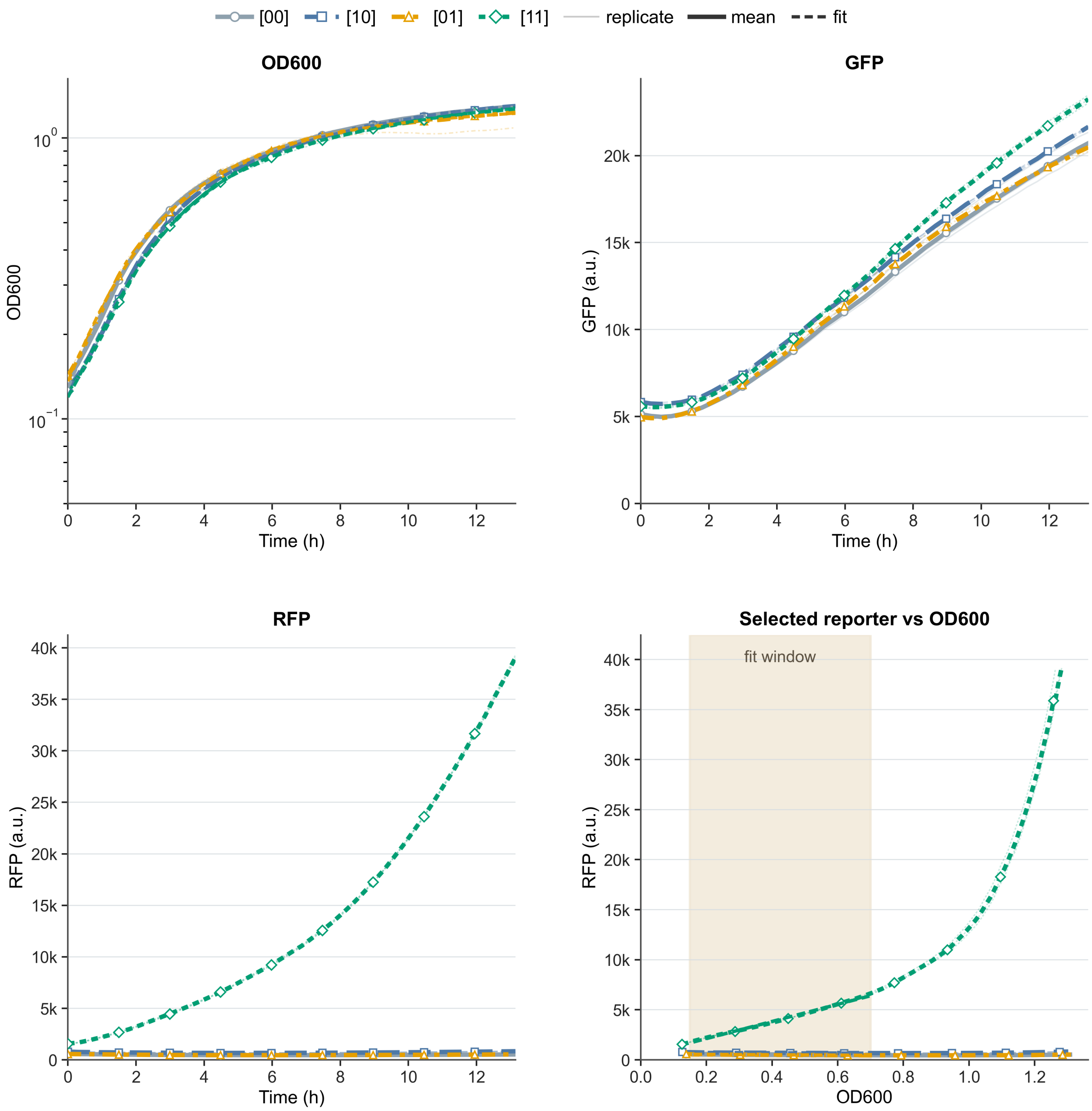

Supplementary Fig. S7 | Dynamics and fit diagnostics for pTet-Bet. The four subpanels show OD_{600} versus time with the growth fit, GFP versus time, RFP versus time, and selected-reporter fluorescence versus OD_{600}. Selected reporter: RFP. Inducer pair, using the source grid label with units in Supplementary Table S6: aTc=0.2; Cho=10000. Biological replicates per state: [00] n = 3, [10] n = 3, [01] n = 3, [11] n = 3. GFP and RFP are independent reporter outputs, not normalisation controls. The fluorescence-versus-OD diagnostic is the panel used to derive the normalised fluorescence for the selected reporter.

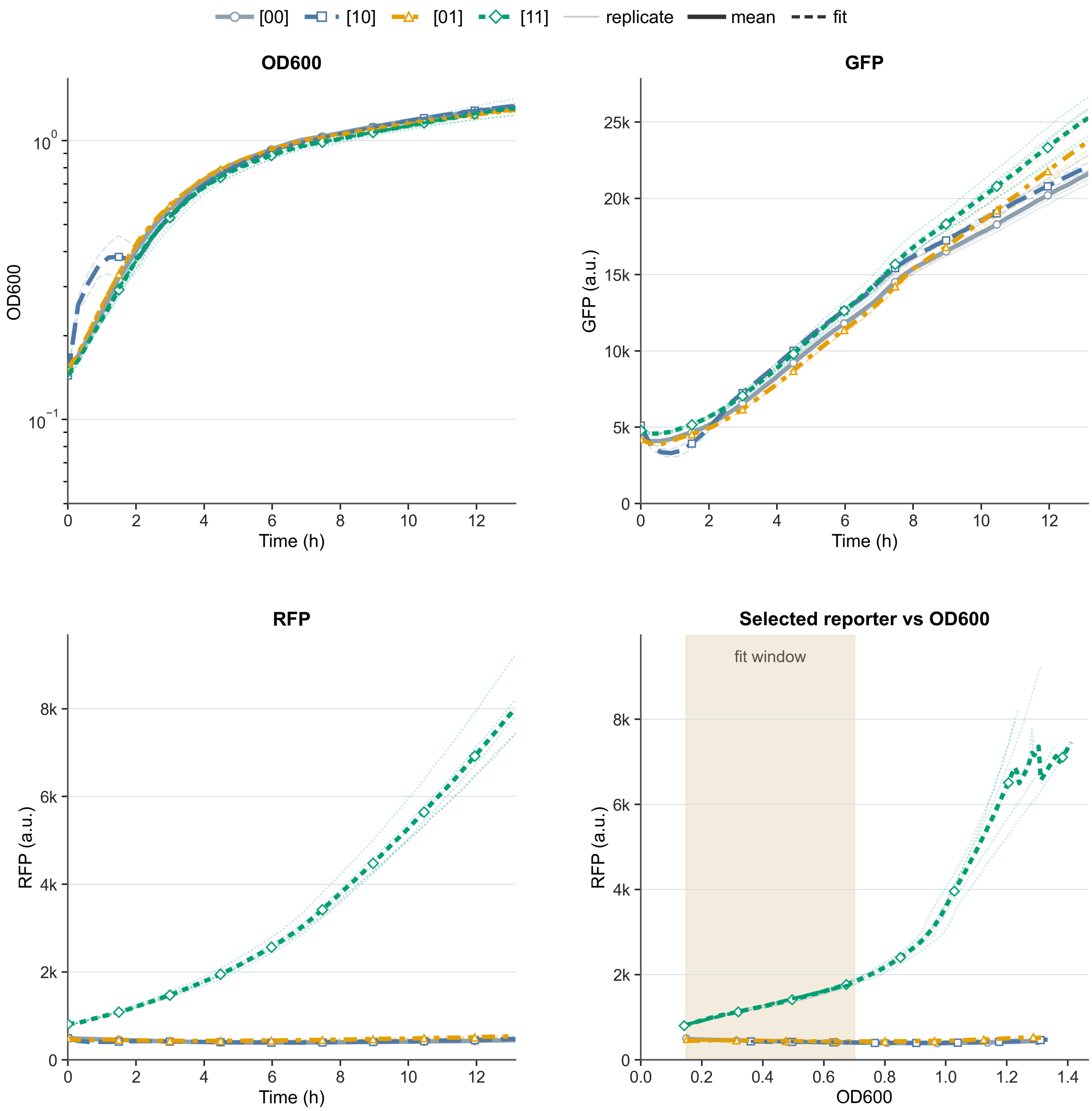

Supplementary Fig. S8 | Dynamics and fit diagnostics for pTet-Ttg. The four subpanels show OD_{600} versus time with the growth fit, GFP versus time, RFP versus time, and selected-reporter fluorescence versus OD_{600}. Selected reporter: RFP. Inducer pair, using the source grid label with units in Supplementary Table S6: aTc=100; Nar=280. Biological replicates per state: [00] n = 4, [10] n = 2, [01] n = 3, [11] n = 6. GFP and RFP are independent reporter outputs, not normalisation controls. The fluorescence-versus-OD diagnostic is the panel used to derive the normalised fluorescence for the selected reporter.

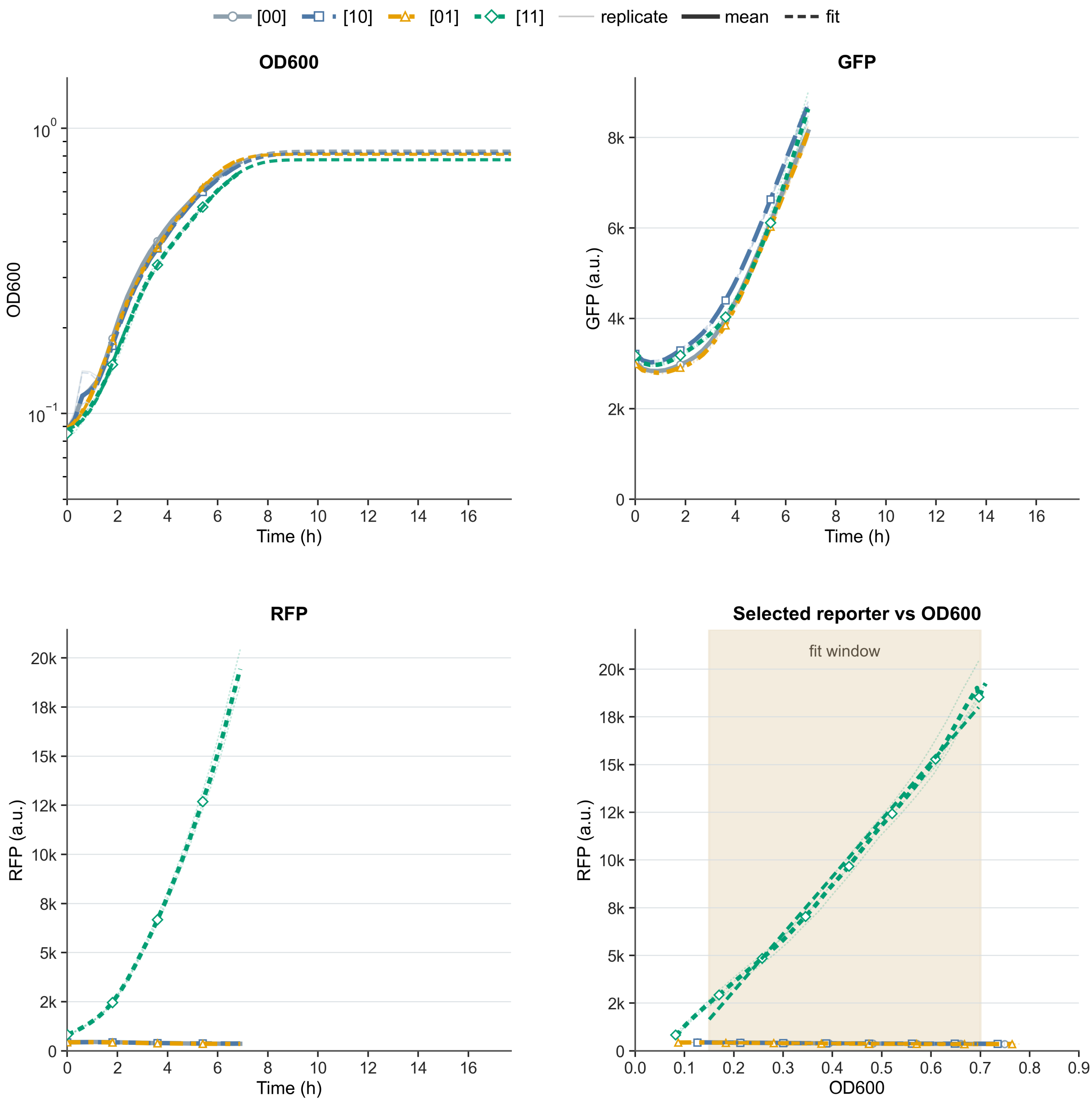

Supplementary Fig. S9 | Dynamics and fit diagnostics for pTet-Van. The four subpanels show OD_{600} versus time with the growth fit, GFP versus time, RFP versus time, and selected-reporter fluorescence versus OD_{600}. Selected reporter: RFP. Inducer pair, using the source grid label with units in Supplementary Table S6: aTc=0.2; Van=100. Biological replicates per state: [00] n = 3, [10] n = 3, [01] n = 3, [11] n = 3. GFP and RFP are independent reporter outputs, not normalisation controls. The fluorescence-versus-OD diagnostic is the panel used to derive the normalised fluorescence for the selected reporter.

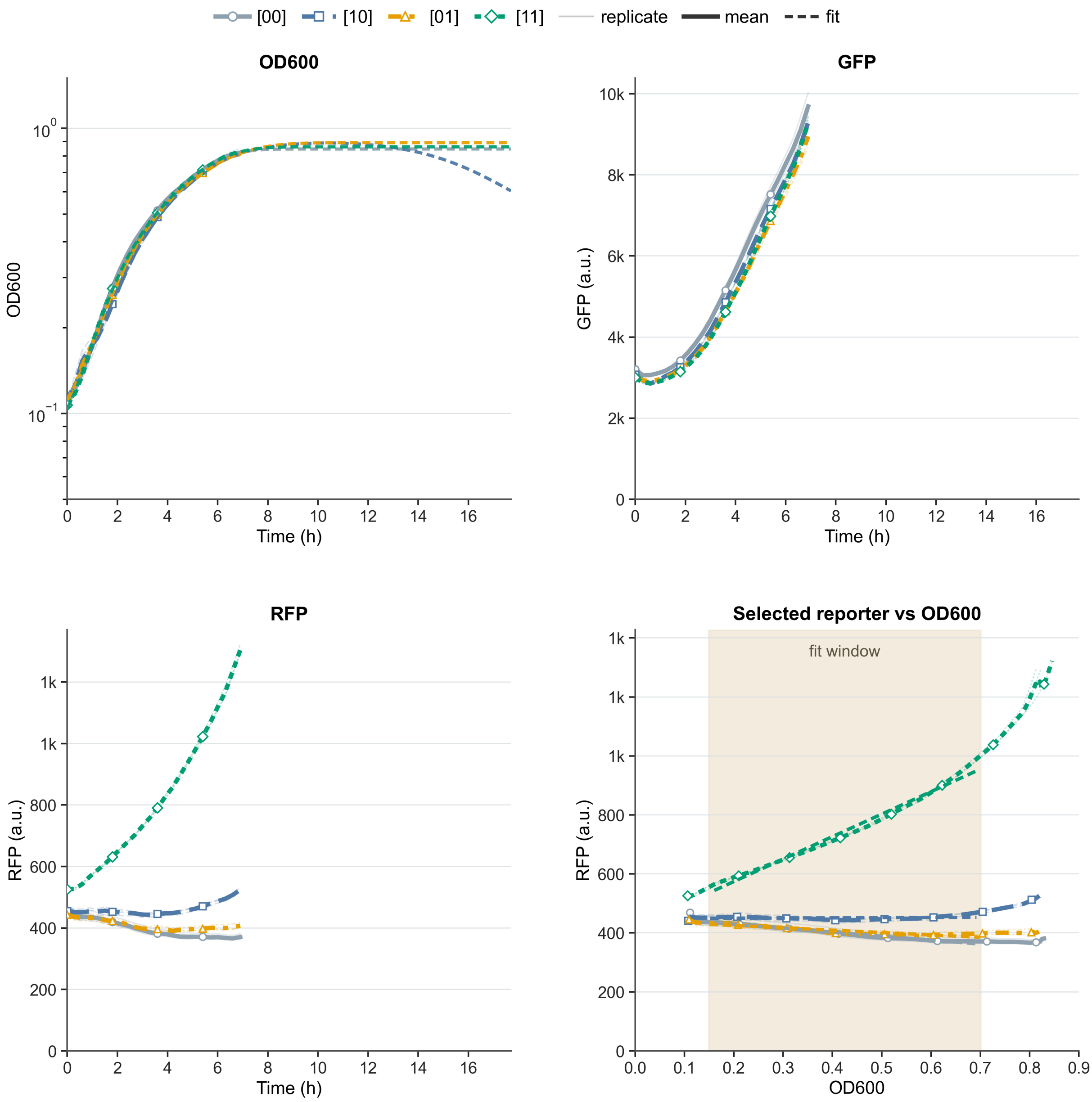

Supplementary Fig. S10 | Dynamics and fit diagnostics for pTtg-Tac. The four subpanels show OD_{600} versus time with the growth fit, GFP versus time, RFP versus time, and selected-reporter fluorescence versus OD_{600}. Selected reporter: RFP. Inducer pair, using the source grid label with units in Supplementary Table S6: Nar=280; IPTG=500. Biological replicates per state: [00] n = 3, [10] n = 3, [01] n = 3, [11] n = 3. GFP and RFP are independent reporter outputs, not normalisation controls. The fluorescence-versus-OD diagnostic is the panel used to derive the normalised fluorescence for the selected reporter.

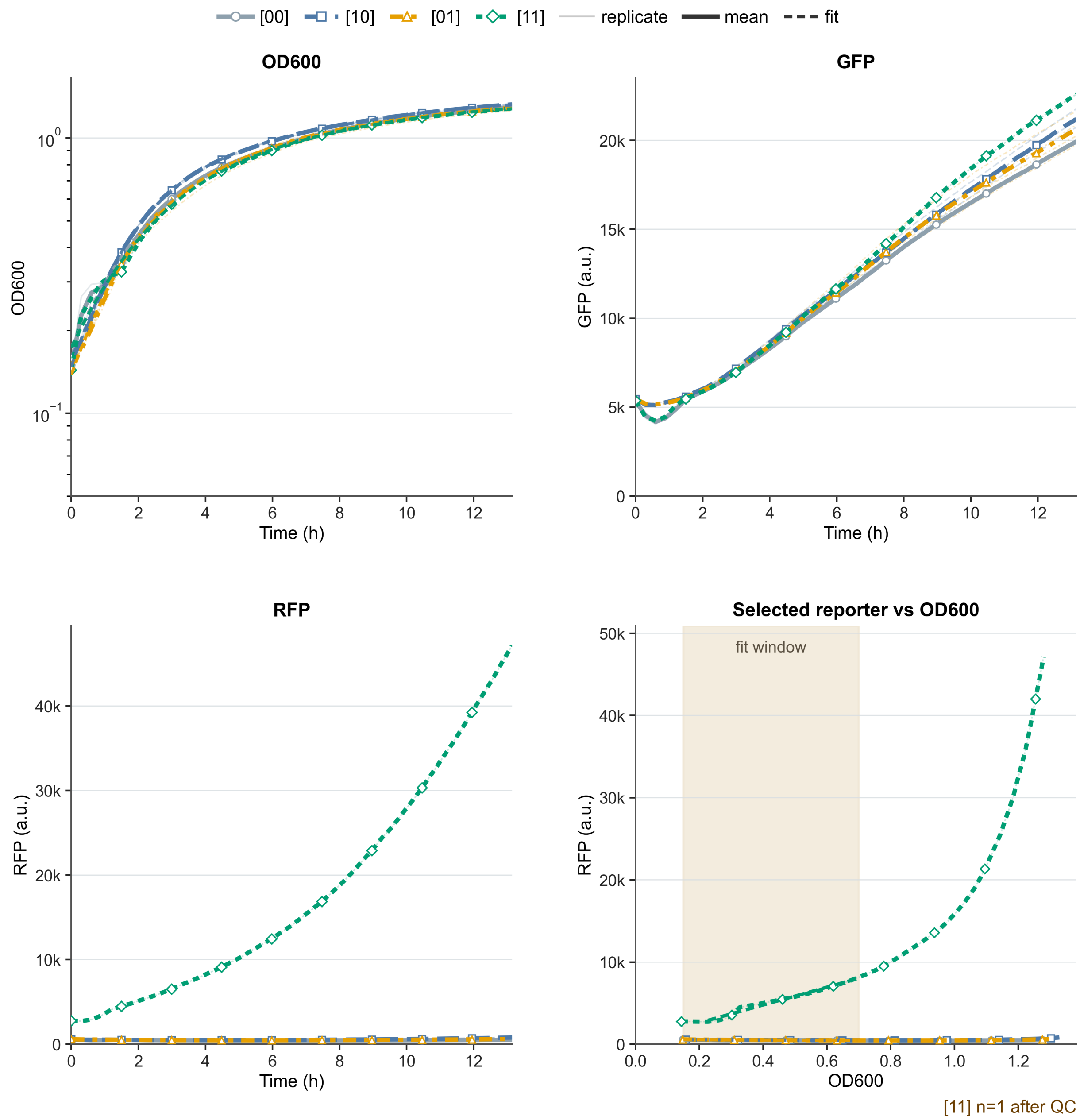

Supplementary Fig. S11 | Dynamics and fit diagnostics for pVan-Tac. The four subpanels show OD_{600} versus time with the growth fit, GFP versus time, RFP versus time, and selected-reporter fluorescence versus OD_{600}. Selected reporter: RFP. Inducer pair, using the source grid label with units in Supplementary Table S6: Van=21; IPTG=1000. Biological replicates per state: [00] n = 3, [10] n = 3, [01] n = 4, [11] n = 1. The [11] output was high, but this row is N.C. because the [11] state has insufficient biological-replicate support after QC. GFP and RFP are independent reporter outputs, not normalisation controls. The fluorescence-versus-OD diagnostic is the panel used to derive the normalised fluorescence for the selected reporter.

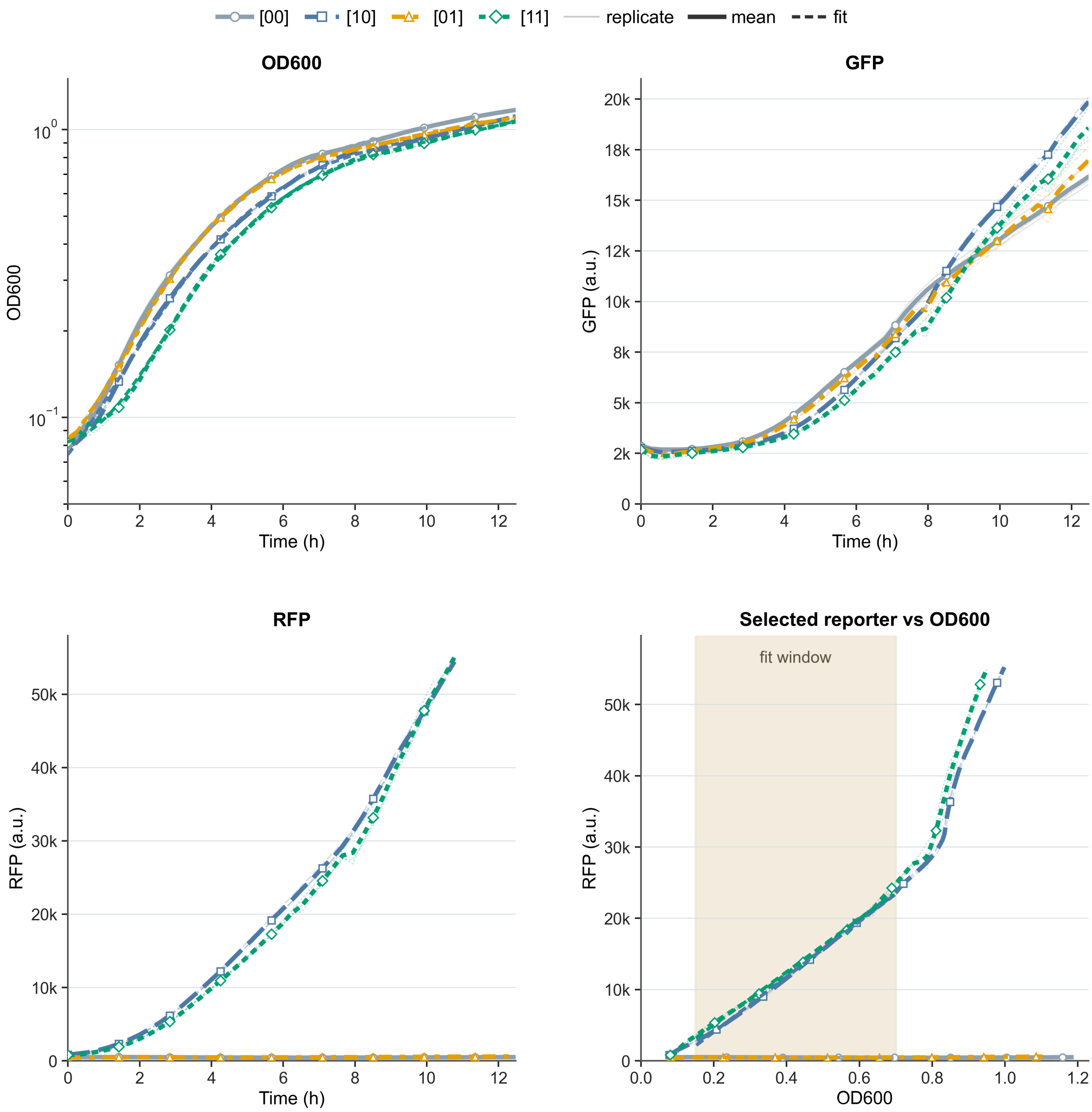

Supplementary Fig. S12 | Dynamics and fit diagnostics for pVan-Ttg. The four subpanels show OD_{600} versus time with the growth fit, GFP versus time, RFP versus time, and selected-reporter fluorescence versus OD_{600}. Selected reporter: RFP. Inducer pair, using the source grid label with units in Supplementary Table S6: Van=100; Nar=1000. Biological replicates per state: [00] n = 3, [10] n = 3, [01] n = 3, [11] n = 3. GFP and RFP are independent reporter outputs, not normalisation controls. The fluorescence-versus-OD diagnostic is the panel used to derive the normalised fluorescence for the selected reporter.

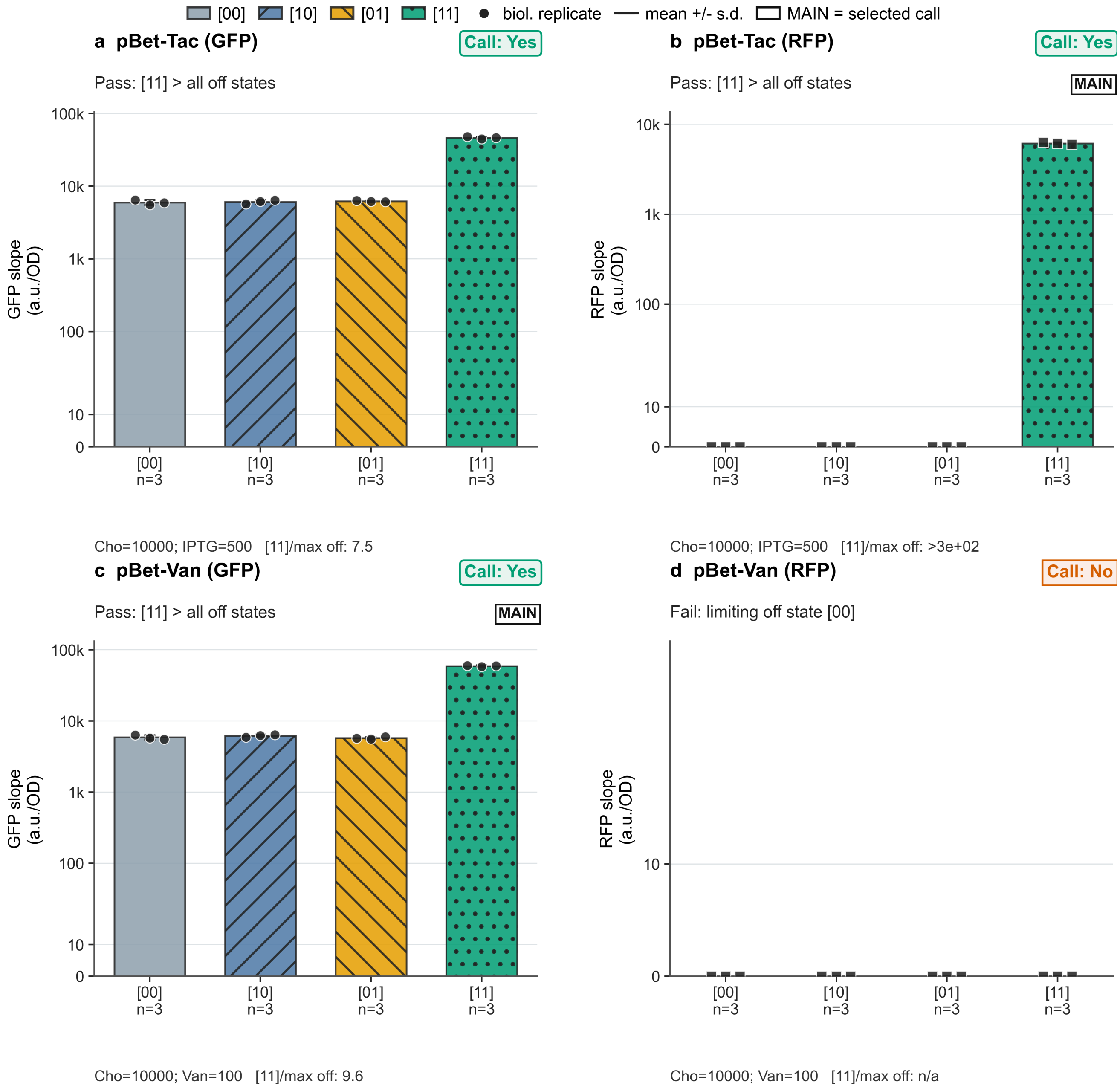

Supplementary Fig. S13 | Reporter-specific truth tables for pBet-derived architectures matching Supplementary Table S2. a, pBet-Tac (GFP), **b,** pBet-Tac (RFP), **c,** pBet-Van (GFP), **d,** pBet-Van (RFP). Bars show biological-replicate mean fluorescence-versus-OD slopes, error bars denote s.d., and symbols show individual biological-replicate values. Bars use fixed [00]/[10]/[01]/[11] state colours and hatching. The MAIN badge marks the reporter row used as the architecture-level representative in the main figures. Call badges show Yes for rows where [11] passes the predefined Holm-adjusted Welch operational screen against all off states, No for rows that fail that screen, and N.C. for rows that cannot be classified because one or more required comparisons lack sufficient biological-replicate support. Limiting-state text identifies the off state that restricts separation, while full Holm-adjusted one-sided Welch P values remain in Supplementary Table S2. The y-axis uses symlog scaling with a linear region from 0 to 20 a.u./OD so zero and low values remain visible. GFP and RFP/mCherry are independent output reporters, not normalisation controls.

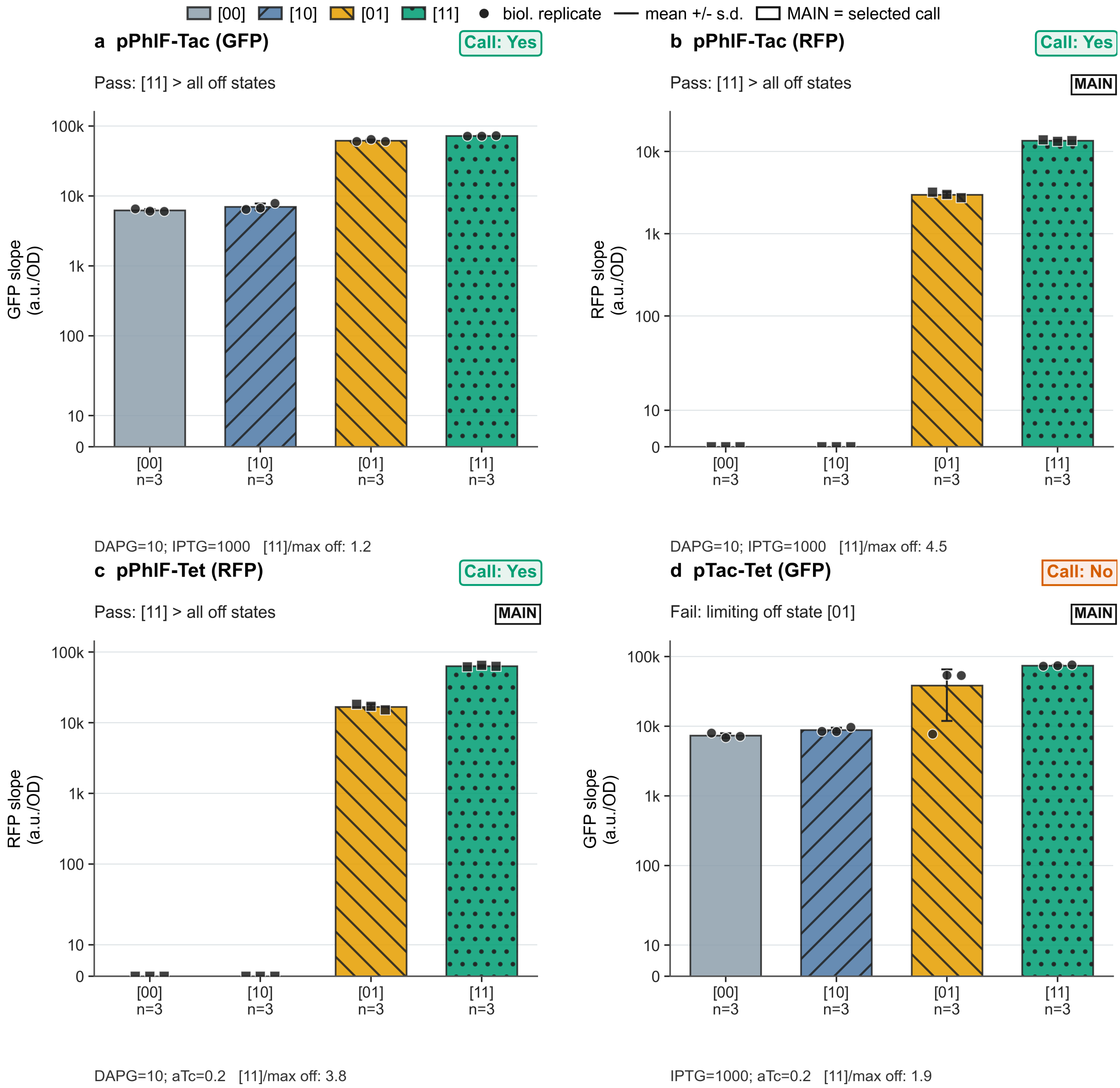

Supplementary Fig. S14 | Reporter-specific truth tables for pPhlF- and pTac-derived architectures matching Supplementary Table S2. a, pPhlF-Tac (GFP), **b,** pPhlF-Tac (RFP), **c,** pPhlF-Tet (RFP/mCherry), **d,** pTac-Tet (GFP). pPhlF-Tac(GFP) is an operational pass but not a practical high-utility gate because the [01] state remains close to [11]. Bars show biological-replicate mean fluorescence-versus-OD slopes, error bars denote s.d., and symbols show individual biological-replicate values. Bars use fixed [00]/[10]/[01]/[11] state colours and hatching. The MAIN badge marks the reporter row used as the architecture-level representative in the main figures. Call badges show Yes for rows where [11] passes the predefined Holm-adjusted Welch operational screen against all off states, No for rows that fail that screen, and N.C. for rows that cannot be classified because one or more required comparisons lack sufficient biological-replicate support. Limiting-state text identifies the off state that restricts separation, while full Holm-adjusted one-sided Welch P values remain in Supplementary Table S2. The y-axis uses symlog scaling with a linear region from 0 to 20 a.u./OD so zero and low values remain visible. GFP and RFP/mCherry are independent output reporters, not normalisation controls.

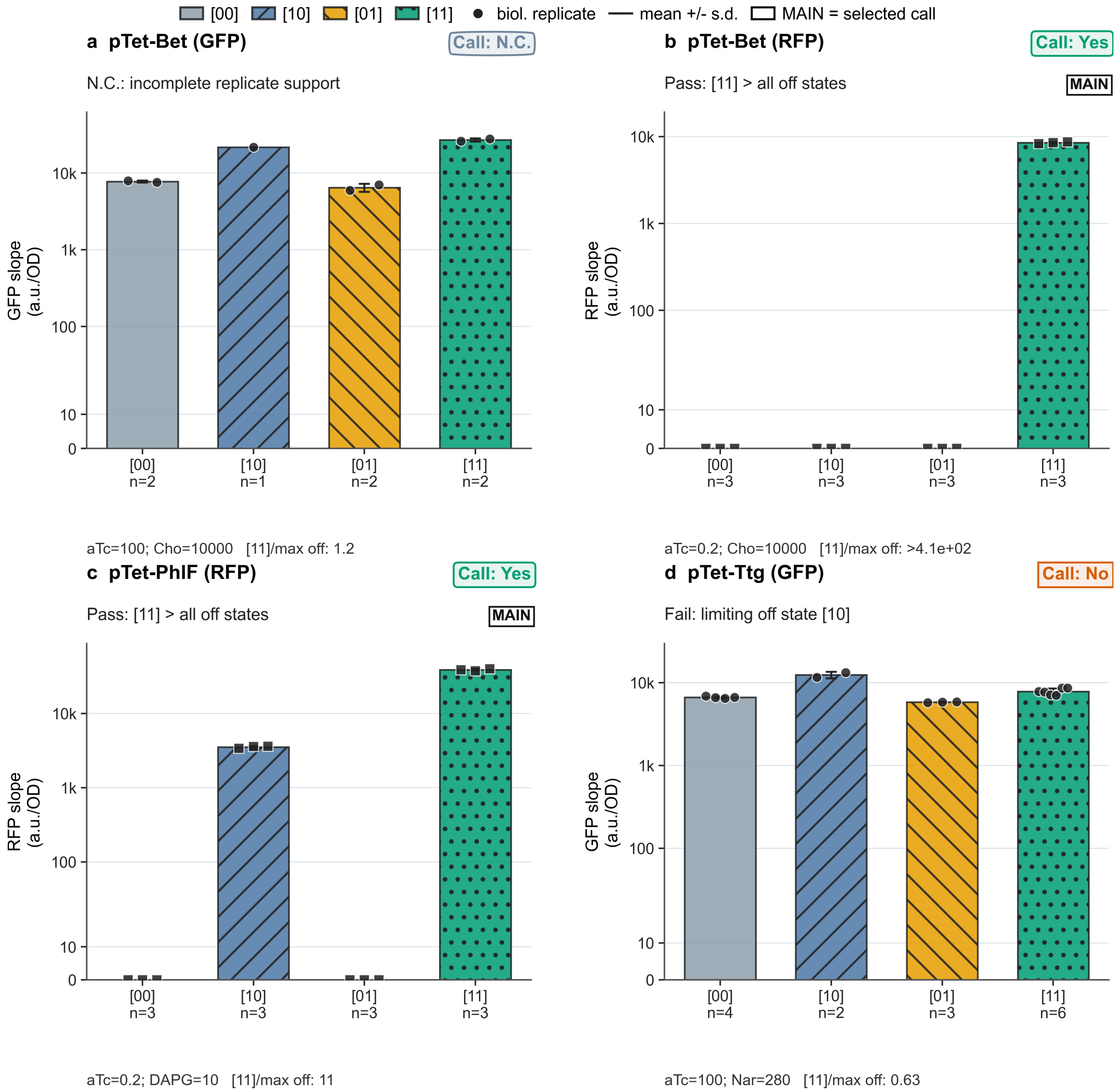

Supplementary Fig. S15 | Reporter-specific truth tables for pTet-derived architectures matching Supplementary Table S2. a, pTet-Bet (GFP), **b,** pTet-Bet (RFP/mCherry), **c,** pTet-PhlF (RFP/mCherry), **d,** pTet-Ttg (GFP). Bars show biological-replicate mean fluorescence-versus-OD slopes, error bars denote s.d., and symbols show individual biological-replicate values. Bars use fixed [00]/[10]/[01]/[11] state colours and hatching. The MAIN badge marks the reporter row used as the architecture-level representative in the main figures. Call badges show Yes for rows where [11] passes the predefined Holm-adjusted Welch operational screen against all off states, No for rows that fail that screen, and N.C. for rows that cannot be classified because one or more required comparisons lack sufficient biological-replicate support. Limiting-state text identifies the off state that restricts separation, while full Holm-adjusted one-sided Welch P values remain in Supplementary Table S2. The y-axis uses symlog scaling with a linear region from 0 to 20 a.u./OD so zero and low values remain visible. GFP and RFP/mCherry are independent output reporters, not normalisation controls.

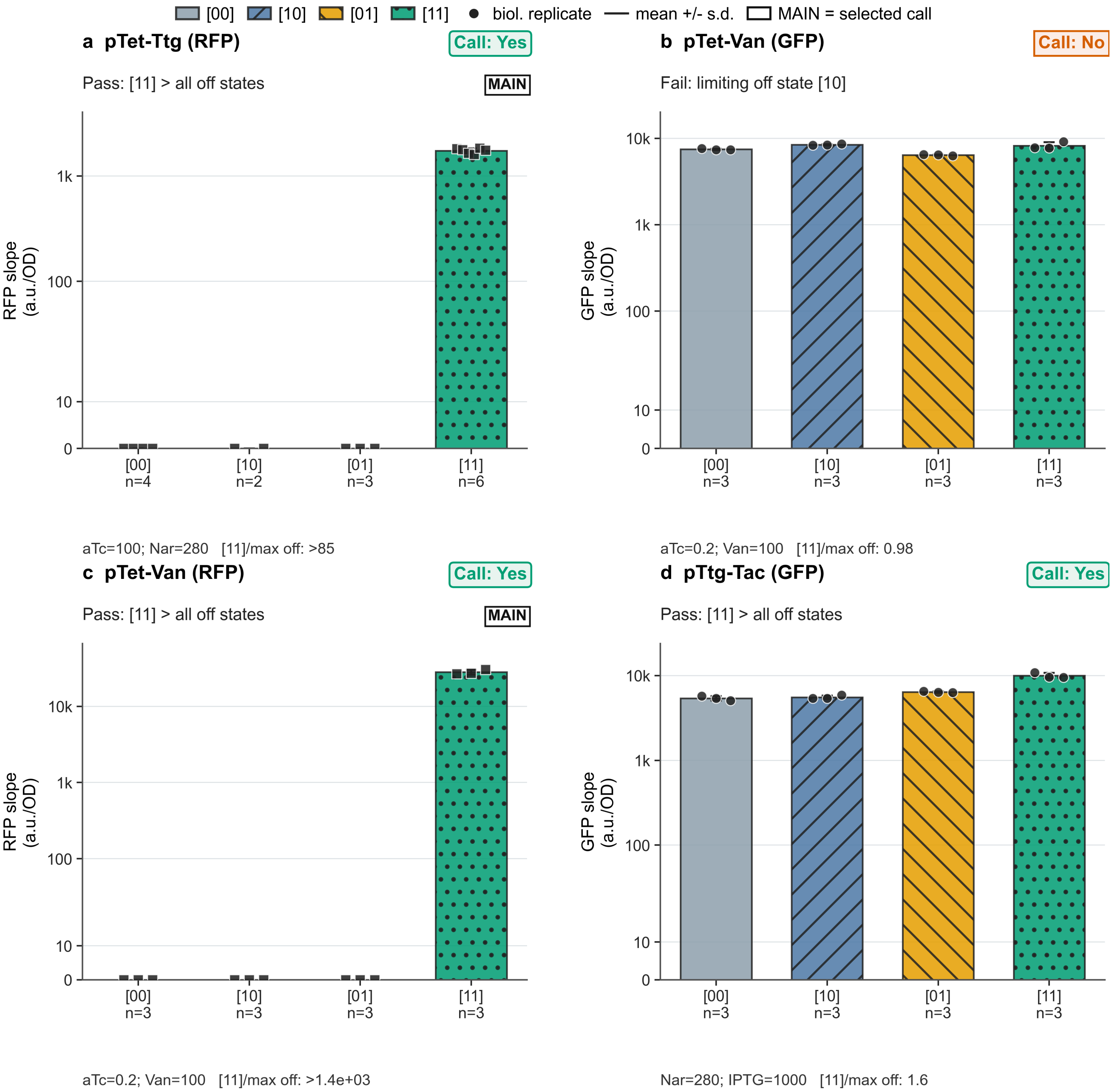
[00] [10] [01] [11] biol. replicate mean +/- s.d. MAIN = selected call
a pTet-Ttg (RFP)
Call: Yes
Pass: [11] > all off states
MAIN
RFP slope (a.u./OD)
1k
100
10
0
[00] n=4
[10] n=2
[01] n=3
[11] n=6
aTc=100; Nar=280 [11]/max off: >85
b pTet-Van (GFP)
Call: No
Fail: limiting off state [10]
GFP slope (a.u./OD)
10k
1k
100
10
0
[00] n=3
[10] n=3
[01] n=3
[11] n=3
aTc=0.2; Van=100 [11]/max off: 0.98
c pTet-Van (RFP)
Call: Yes
Pass: [11] > all off states
MAIN
RFP slope (a.u./OD)
10k
1k
100
10
0
[00] n=3
[10] n=3
[01] n=3
[11] n=3
aTc=0.2; Van=100 [11]/max off: >1.4e+03
d pTtg-Tac (GFP)
Call: Yes
Pass: [11] > all off states
GFP slope (a.u./OD)
10k
1k
100
10
0
[00] n=3
[10] n=3
[01] n=3
[11] n=3
Nar=280; IPTG=1000 [11]/max off: 1.6

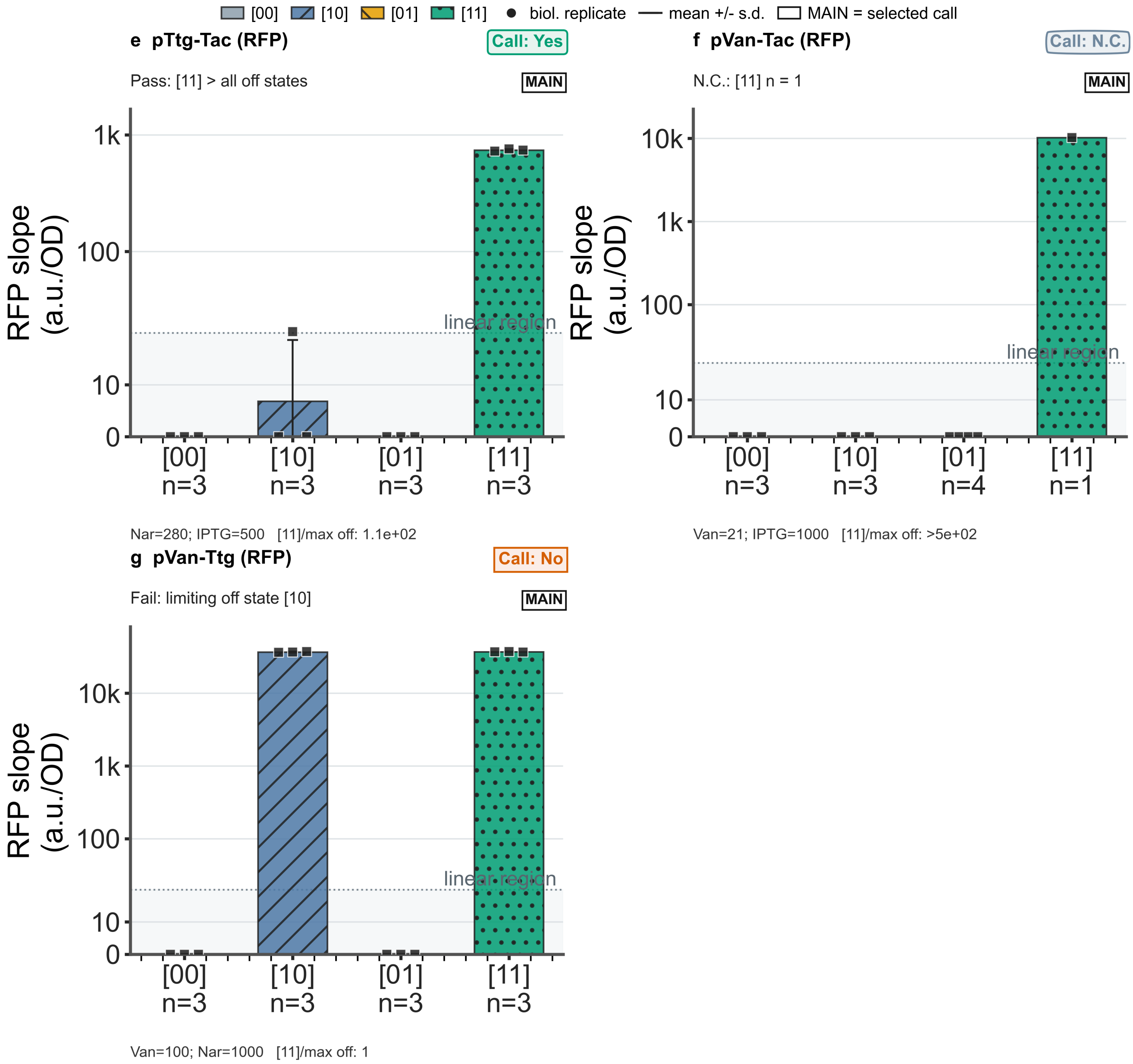

Supplementary Fig. S16 | Reporter-specific truth tables for additional pTet-, pTtg- and pVan-derived architectures matching Supplementary Table S2. a, pTet-Ttg (RFP/mCherry), **b,** pTet-Van (GFP), **c,** pTet-Van (RFP/mCherry), **d,** pTtg-Tac (GFP), **e,** pTtg-Tac (RFP/mCherry), **f,** pVan-Tac (RFP/mCherry), **g,** pVan-Ttg (RFP/mCherry). Bars show biological-replicate mean fluorescence-versus-OD slopes, error bars denote s.d., and symbols show individual biological-replicate values. Bars use fixed [00]/[10]/[01]/[11] state colours and hatching. The MAIN badge marks the reporter row used as the architecture-level representative in the main figures. Call badges show Yes for rows where [11] passes the predefined Holm-adjusted Welch operational screen against all off states, No for rows that fail that screen, and N.C. for rows that cannot be classified because one or more required comparisons lack sufficient biological-replicate support. Limiting-state text identifies the off state that restricts separation, while full Holm-adjusted one-sided Welch P values remain in Supplementary Table S2. The y-axis uses symlog scaling with a linear region from 0 to 20 a.u./OD so zero and low values remain visible. GFP and RFP/mCherry are independent output reporters, not normalisation controls. pVan-Tac is N.C. because [11] has n = 1 after QC. pVan-Ttg is a No/failed truth table with [10] as the limiting off state.

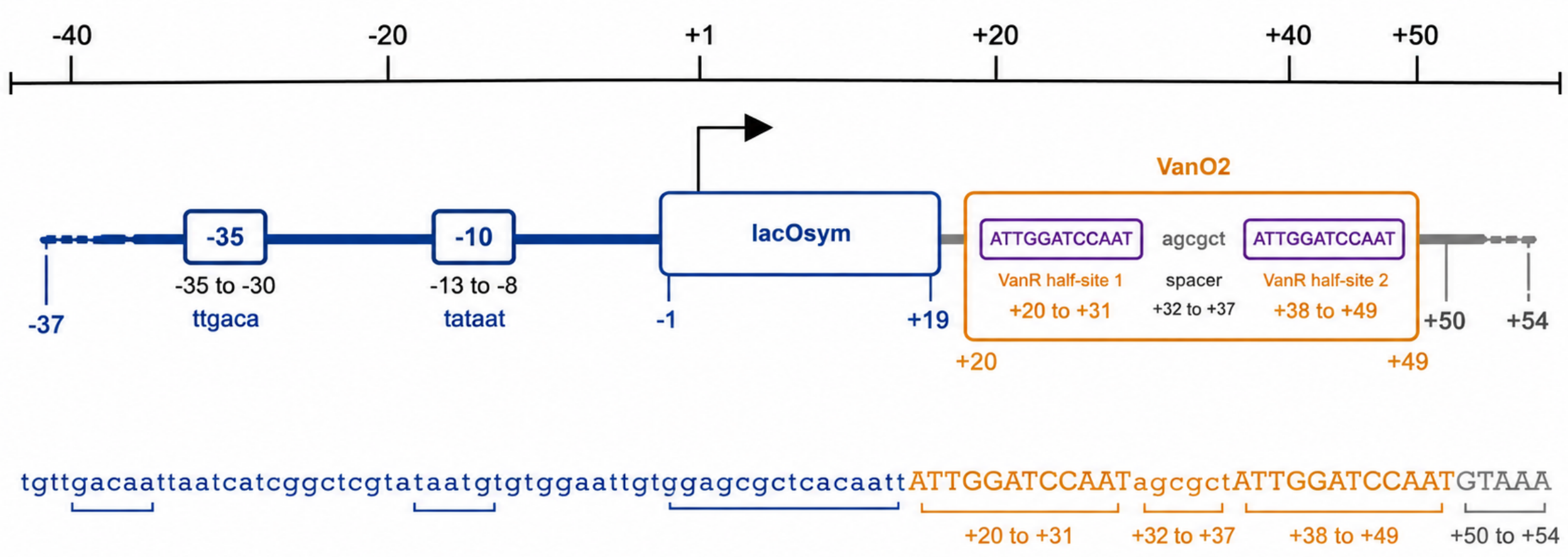

Feature	Coordinates
pTac template	-37 to +19
lacOsym	-1 to +19
VanO2	+20 to +49
downstream flank	+50 to +54

Supplementary Fig. S17 | Architecture of the experimentally tested pTac-Van combinatorial promoter. The compact promoter region is drawn to scale and positions are reported relative to the transcription start site (+1). The pTac template, lacOsym site and VanO2 site are coloured consistently with main-text Fig. 1b and Supplementary Table S7. This architecture was experimentally tested and is documented in Supplementary Table S7, but it was not included in the curated main-text architecture set shown in main-text Fig. 1b.

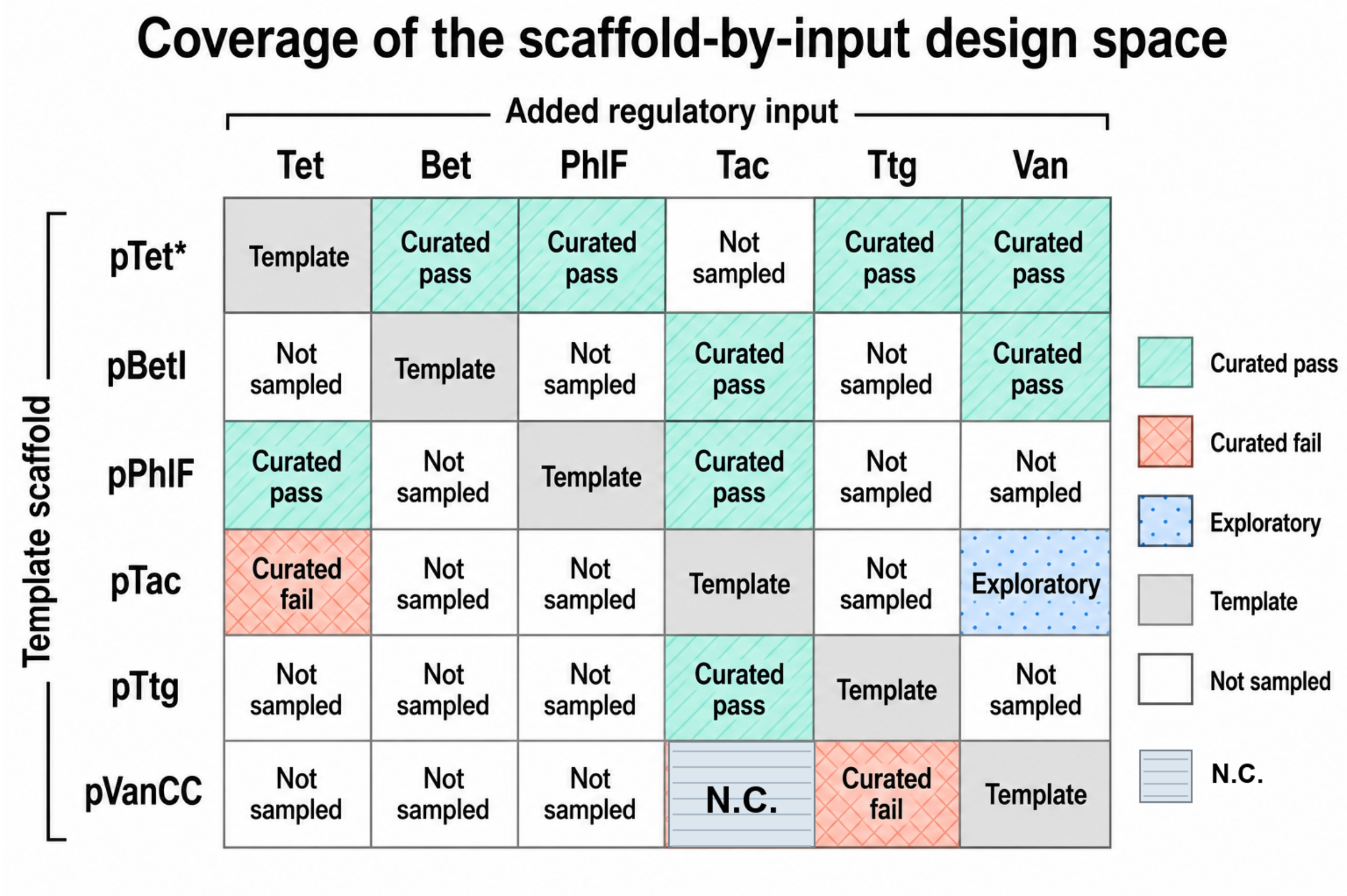

Supplementary Fig. S18 | Coverage of the scaffold-by-input design space used in this study. The matrix uses colour and text labels to distinguish curated architectures that passed the operational screen (green), curated architectures that failed (red), the not-classifiable pVan-Tac case (grey-blue N.C.), the exploratory pTac-Van construct (blue), template-only entries (grey), and scaffold-input combinations that were not sampled in the present benchmark (white).

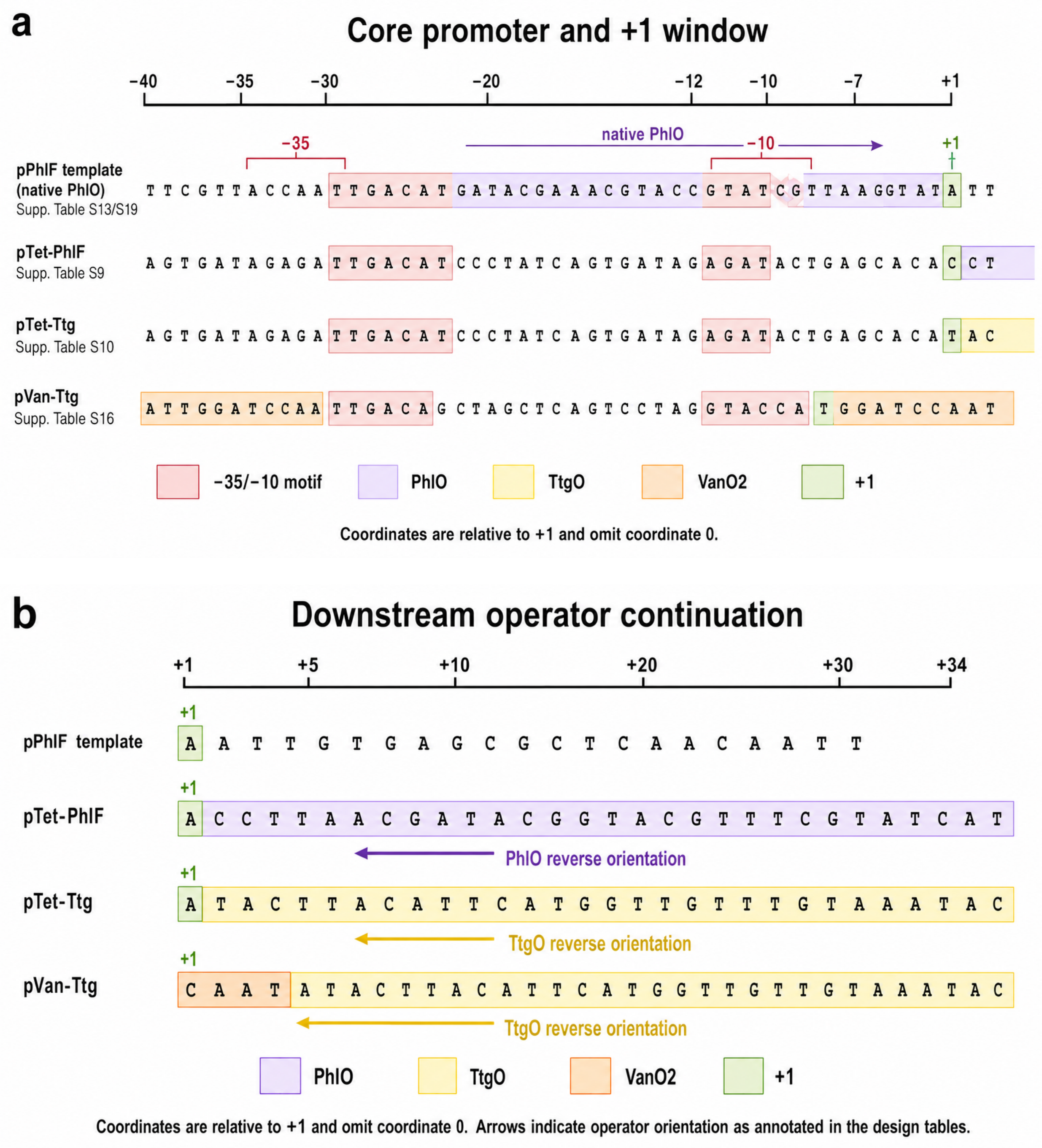

Supplementary Fig. S19 | Sequence alignment around the -10 region and +1 site in reverse-operator examples. a, Core-promoter/+1 window. **b,** Downstream operator continuation. Rows show pPhlF template/native PhlO, pTet-PhlF, pTet-Ttg and pVan-Ttg. DNA bases are monospaced. Coordinates are relative to +1 and omit coordinate 0. Colours mark PhlO (purple), TtgO (yellow), VanO2 (orange), annotated -35/-10 motifs (pink) and +1 (green). Reverse orientation is indicated for inserted PhlO and TtgO. The figure illustrates sequence-aware placement used to avoid promoter-like motif arrangements in compact downstream-operator designs. It does not demonstrate that orientation caused promoter behaviour.

Supplementary Tables

Distributed file	Contents
source_data.xlsx	Source-data workbook distributed with the submission. It contains source data for retained main and supplementary figures and tables.
code_ocean_scripts.zip	Python scripts for reproducing the processed analyses and regenerated quantitative charts from the submitted source-data workbook.
snapgene_files.zip	SnapGene DNA files for the promoter-reporter plasmid constructs.

Supplementary Table S1 | Distributed source-data and reproducibility files.

a

Promoter	Reporter	Template	Added input	Template inducer	Added inducer	Inducer pair	Selected main call	Operational call	Engineering Utility
pBet-Tac	GFP	pBetI	Tac	Cho	IPTG	Cho=10000; IPTG=500	No	Yes	True
pBet-Tac	RFP	pBetI	Tac	Cho	IPTG	Cho=10000; IPTG=500	Yes	Yes	True
pBet-Van	GFP	pBetI	Van	Cho	Van	Cho=10000; Van=100	Yes	Yes	True
pBet-Van	RFP	pBetI	Van	Cho	Van	Cho=10000; Van=100	No	No	False
pPhlF-Tac	GFP	pPhlF	Tac	DAPG	IPTG	DAPG=10; IPTG=1000	No	Yes	False
pPhlF-Tac	RFP	pPhlF	Tac	DAPG	IPTG	DAPG=10; IPTG=1000	Yes	Yes	True
pPhlF-Tet	RFP	pPhlF	Tet	DAPG	aTc	DAPG=10; aTc=0.2	Yes	Yes	True
pTac-Tet	GFP	pTac	Tet	IPTG	aTc	IPTG=1000; aTc=0.2	Yes	No	False
pTet-Bet	GFP	pTet*	Bet	aTc	Cho	aTc=100; Cho=10000	No	N.C.	False
pTet-Bet	RFP	pTet*	Bet	aTc	Cho	aTc=0.2; Cho=10000	Yes	Yes	True
pTet-PhlF	RFP	pTet*	PhlF	aTc	DAPG	aTc=0.2; DAPG=10	Yes	Yes	True
pTet-Ttg	GFP	pTet*	Ttg	aTc	Nar	aTc=100; Nar=280	No	No	False
pTet-Ttg	RFP	pTet*	Ttg	aTc	Nar	aTc=100; Nar=280	Yes	Yes	True
pTet-Van	GFP	pTet*	Van	aTc	Van	aTc=0.2; Van=100	No	No	False
pTet-Van	RFP	pTet*	Van	aTc	Van	aTc=0.2; Van=100	Yes	Yes	True
pTtg-Tac	GFP	pTtg	Tac	Nar	IPTG	Nar=280; IPTG=1000	No	Yes	False
pTtg-Tac	RFP	pTtg	Tac	Nar	IPTG	Nar=280; IPTG=500	Yes	Yes	True
pVan-Tac	RFP	pVanCC	Tac	Van	IPTG	Van=21; IPTG=1000	Yes	N.C.	False
pVan-Ttg	RFP	pVanCC	Ttg	Van	Nar	Van=100; Nar=1000	Yes	No	False

b

Promoter	Reporter	[00] mean	[10] mean	[01] mean	[11] mean
pBet-Tac	GFP	5973 (6472)	6075 (6858)	6206 (6398)	46516 (5710)
pBet-Tac	RFP	0.000 (0.000)	0.000 (0.000)	0.000 (0.000)	6139 (0.000)
pBet-Van	GFP	5876	6180	5741	59190
pBet-Van	RFP	0.000	0.000	0.000	0.000
pPhlF-Tac	GFP	6200 (5807)	6986 (5361)	61486 (5746)	71953 (7336)
pPhlF-Tac	RFP	0.000 (0.000)	0.000 (0.000)	2988 (0.000)	13564 (0.000)
pPhlF-Tet	RFP	0.000	0.000	16800	63107

pTac-Tet	GFP	7339	8818	38428	74064
pTet-Bet	GFP	7753 (5253)	21701 (5957)	6455 (6100)	26924 (6583)
pTet-Bet	RFP	0.000 (0.000)	0.000 (1063)	0.000 (0.000)	8479 (2188)
pTet-PhlF	RFP	0.000	3517	0.000	38690
pTet-Ttg	GFP	6649	12377	5806	7813
pTet-Ttg	RFP	0.000	0.000	0.000	1740
pTet-Van	GFP	7451	8425	6395	8216
pTet-Van	RFP	0.000	0.000	0.000	28030
pTtg-Tac	GFP	5386 (6858)	5547 (6899)	6389 (6304)	10002 (6092)
pTtg-Tac	RFP	0.000 (0.000)	6.831 (0.000)	0.000 (0.000)	739.87 (0.000)
pVan-Tac	RFP	0.000	0.000	0.000	10256
pVan-Ttg	RFP	0.000	36787	0.000	36976

c

Promoter	Reporter	[00] sd	[10] sd	[01] sd	[11] sd	n [00]	n [10]	n [01]	n [11]
pBet-Tac	GFP	465.69	357.55	141.36	1651	3	3	3	3
pBet-Tac	RFP	0.000	0.000	0.000	143.08	3	3	3	3
pBet-Van	GFP	406.05	252.59	217.03	1099	3	3	3	3
pBet-Van	RFP	0.000	0.000	0.000	0.000	3	3	3	3
pPhlF-Tac	GFP	279.05	761.17	2243	678.67	3	3	3	3
pPhlF-Tac	RFP	0.000	0.000	228.75	258.88	3	3	3	3
pPhlF-Tet	RFP	0.000	0.000	1509	1653	3	3	3	3
pTac-Tet	GFP	585.32	691.41	26605	1406	3	3	3	3
pTet-Bet	GFP	245.17	0.000	761.93	1297	2	1	2	2
pTet-Bet	RFP	0.000	0.000	0.000	204.60	3	3	3	3
pTet-PhlF	RFP	0.000	122.34	0.000	1190	3	3	3	3
pTet-Ttg	GFP	189.48	1142	74.51	679.44	4	2	3	6
pTet-Ttg	RFP	0.000	0.000	0.000	94.78	4	2	3	6
pTet-Van	GFP	129.07	139.60	99.68	785.88	3	3	3	3
pTet-Van	RFP	0.000	0.000	0.000	2023	3	3	3	3
pTtg-Tac	GFP	334.90	280.64	129.11	754.13	3	3	3	3
pTtg-Tac	RFP	0.000	11.83	0.000	15.17	3	3	3	3
pVan-Tac	RFP	0.000	0.000	0.000	0.000	3	3	4	1
pVan-Ttg	RFP	0.000	509.59	0.000	236.54	3	3	3	3

d

Promoter	Reporter	Max off	Highest off	[11]/max off	Lower bound	Holm P [11]>[00]	Holm P [11]>[10]	Holm P [11]>[01]
pBet-Tac	GFP	6206	[01]	7.495	No	<0.001	<0.001	<0.001
pBet-Tac	RFP	0.000	[00]	>299.55	Yes	<0.001	<0.001	<0.001
pBet-Van	GFP	6180	[10]	9.578	No	<0.001	<0.001	<0.001
pBet-Van	RFP	0.000	[00]		Yes	1.000	1.000	1.000
pPhlF-Tac	GFP	61486	[01]	1.170	No	<0.001	<0.001	0.005
pPhlF-Tac	RFP	2988	[01]	4.539	No	<0.001	<0.001	<0.001

pPhlF-Tet	RFP	16800	[01]	3.756	No	<0.001	<0.001	<0.001
pTac-Tet	GFP	38428	[01]	1.927	No	<0.001	<0.001	0.073
pTet-Bet	GFP	21701	[10]	1.241	No			
pTet-Bet	RFP	0.000	[00]	>413.74	Yes	<0.001	<0.001	<0.001
pTet-PhlF	RFP	3517	[10]	11.00	No	<0.001	<0.001	<0.001
pTet-Ttg	GFP	12377	[10]	0.631	No	0.007	0.959	0.001
pTet-Ttg	RFP	0.000	[00]	>84.88	Yes	<0.001	<0.001	<0.001
pTet-Van	GFP	8425	[10]	0.975	No	0.232	0.654	0.082
pTet-Van	RFP	0.000	[00]	>1368	Yes	0.003	0.003	0.003
pTtg-Tac	GFP	6389	[01]	1.566	No	0.005	0.005	0.006
pTtg-Tac	RFP	6.831	[10]	108.30	No	<0.001	<0.001	<0.001
pVan-Tac	RFP	0.000	[00]	>500.44	Yes			
pVan-Ttg	RFP	36787	[10]	1.005	No	<0.001	0.302	<0.001

Supplementary Table S2 | Statistics for all 19 curated two-input combinatorial promoter-reporter truth tables. The table reports all curated reporter-specific truth tables rather than only the selected architecture-level rows. **a,** Construct identity, operational classification and engineering-utility flag. **b,** State means; parenthetical values report same-channel alternate-reporter diagnostics where an independent matched alternate reporter context is available. **c,** State standard deviations and biological replicate counts. **d,** Off-state ratio and Holm-adjusted comparisons. The selected main call column identifies the reporter used for the main architecture-level call. Ratios with non-positive maximum off-state means are reported as lower bounds rather than infinity. N.C. or not_classifiable indicates that one or more required state comparisons lacked sufficient biological-replicate support.

Promoter	Reporter	Reason if not selected
pBet-Tac	GFP	not selected as architecture-level representative
pBet-Tac	RFP	
pBet-Van	GFP	
pBet-Van	RFP	not selected as architecture-level representative
pPhlF-Tac	GFP	not selected as architecture-level representative
pPhlF-Tac	RFP	
pPhlF-Tet	RFP	
pTac-Tet	GFP	
pTet-Bet	GFP	not selected as architecture-level representative
pTet-Bet	RFP	
pTet-PhlF	RFP	
pTet-Ttg	GFP	not selected as architecture-level representative
pTet-Ttg	RFP	
pTet-Van	GFP	not selected as architecture-level representative
pTet-Van	RFP	
pTtg-Tac	GFP	not selected as architecture-level representative
pTtg-Tac	RFP	
pVan-Tac	RFP	
pVan-Ttg	RFP	

Supplementary Table S3 | Selection notes and provenance.

#	Primer name	Sequence
1	AND pTet-Ttg (aTc+NAR)151 FW	agcacATACTTACATTCATGGTTGTTTGTAAATACACagctgtcaccggatgtgctttc
	AND pTet-Ttg (aTc+NAR)152 RW	gctGTGTATTTACAAACAACCATGAATGTAAGTATgtgctcagtatctctatcactgata gggatg
2	AND pVan-Ttg (Van+NAR) 155 FW	ATACTTACATTCATGGTTGTTTGTAAATACTGTAAACagctgtcaccggatgtgctttc
	AND pVan-Ttg (Van+NAR) 156 RW	TTTACATTGGATCCAATagcgctATTGGATCCAATaattgtgagcgctcacaattccaca c
3	AND pBetI-Tac (cho-IPTG)161) FW	ataatgctagcaattgtgagcgctcacaattAAGATCGGGTGTAAACagctgtcac
	AND pBetI-Tac (cho-IPTG)162) FW	CCCGATCTTaattgtgagcgctcacaattgctagcattatattgaacgtccaatcaattg
4	AND pPhlF-Tac(DAPG- IPTG)165 FW	gtatcgttaaggtaattgtgagcgctcacaattTCGGGTGTAAACagctgtcaccg
	AND pPhlF-Tac(DAPG- IPTG)166 RW	CACCCGAaattgtgagcgctcacaattaccttaacgatacggtacgtttcgtatca
5	AND pTtg-pTac (NAR-IPTG)167 FW	tatattccttagcaaaattgtgagcgctcacaattGGGTGTAAACagctgtcaccggat
	AND pTtg-pTac (NAR-IPTG)168 FW	CACCCaattgtgagcgctcacaattttgctaaggaatatacttacattcatggttgtttg
6	AND pVan-Tac (Van-IPTG)169 FW	tggatccaataattgtgagcgctcacaattTAAGATCGGGTGTAAACagctgtcac
	AND pVan-Tac (Van-IPTG)170 FW	CCCGATCTTAaattgtgagcgctcacaattattggatccaatggtacctaggactg
7	AND pTet -Bet (aTc-Cho)173 FW	atccggtgacagctGTTTACACCCattgattggacgttcaatataagtgctcagtatc
	AND pTet- Bet (aTc-Cho)174 RW	TACACCCattgattggacgttcaatataagtgctcagtatctctatcactgatagggat
8	AND pTet-PhiF (aTc-DAPG)175 FW	ctgagcacaccttaacgatacggtacgtttcgtatcatACagctgtcaccggatgtgctt tc
	AND pTet-PhiF (aTc-DAPG)176 RW	GTatgatacgaaacgtaccgtatcgttaaggtgtgctcagtatctctatcactgataggg atg
9	AND pTet- Van (aTc-VAN)177 FW	gagcacATTGGATCCAATagcgctATTGGATCCAATACagctgtcaccggatgtgctttc
	AND pTet- Van (aTc-VAN)178 RW	ctGTATTGGATCCAATagcgctATTGGATCCAATgtgctcagtatctctatcactgatag ggatg
10	AND pTac-Van (IPTG+VAN)179 FW	acaattATTGGATCCAATagcgctATTGGATCCAATGTAAACagctgtcaccggatgtgc
	AND pTac-Van (IPTG+VAN)180 RW	TTACATTGGATCCAATagcgctATTGGATCCAATaattgtgagcgctcacaattccacac
11	AND pTac-Tet (IPTG-aTc)194 FW	ctatcagtgatagagaTTGTGAGCGGATAACAAagctgtcaccggatgtgctttcc
	AND pTac-Tet (IPTG-aTc)194 FW	gacagctTTGTTATCCGCTCACAAtctctatcactgatagggaaattgtgagcgc

Supplementary Table S4 | Primers used in this work.

Promoter	Sequence
pTac-Tet	tgttgacaattaatcatcggctcgtataatgtgtggaattgtgagcgctcacaatttccctatcagtgatagagaTAAGATCGGGTGTAAA
pTac-Van	tgttgacaattaatcatcggctcgtataatgtgtggaattgtgagcgctcacaattATTGGATCCAATagcgctATTGGATCCAATGTAAA
pTet-Van	ttttcagcaggacgcactgacctccctatcagtgatagagattgacatccctatcagtgatagagatactgagcacATTGGATCCAATagcgctATTGGATCCAATA
pTet-Bet	ttttcagcaggacgcactgacctccctatcagtgatagagattgacatccctatcagtgatagagatactgagcacttatattgaacgtccaatcaatGGGTGTAAA
pTet-Ttg	ttttcagcaggacgcactgacctccctatcagtgatagagattgacatccctatcagtgatagagatactgagcacATACTTACATTCATGGTTGTTTGTAAATAC
pTet-PhlF	ttttcagcaggacgcactgacctccctatcagtgatagagattgacatccctatcagtgatagagatactgagcacaccttaacgatacggtacgtttcgtatcatA
pVan-Ttg	ttggatccaattgacagctagctcagtcctaggtaccattggatccaatATACTTACATTCATGGTTGTTTGTAAATACTGTAAA
pVan-Tac	attggatccaattgacagctagctcagtcctaggtaccattggatccaataattgtgagcgctcacaattTAAGATCGGGTGTAAA
pBet-Tac	agcgcgggtgagagggattcgttaccaatagacaattgattggacgttcaatataatgctagcaattgtgagcgctcacaattAAGATCGGGTGTAAA
pTtg-Tac	cacccagcagtatttacaaacaaccatgaatgtaagtatattccttagcaaaattgtgagcgctcacaattGGGTGTAAA
pPhlF-Tac	cgacgtacggtggaatctgattcgttaccaattgacatgatacgaaacgtaccgtatcgttaaggtaattgtgagcgctcacaattTCGGGTGTAAA

Supplementary Table S5 | Promoter sequences.

a

Inducer	Full name	Unit used in concentration table
DAPG	2,4-diacetylphloroglucinol	µM
IPTG	isopropyl β-D-1-thiogalactopyranoside	µM
aTc	anhydrotetracycline hydrochloride	0.2 µM or 100 ng mL−1, matching the source grid label
Cho	choline chloride	µM
Nar	naringenin	µM
Van	vanillic acid	µM

b

Promoter-reporter and source grid label	[10] first/template input ON	[01] second/added input ON	[11] both inputs ON
pBet-Tac (GFP) Cho=10000; IPTG=500	pBetI input; Cho 10000 µM	Tac input; IPTG 500 µM	Cho 10000 µM + IPTG 500 µM
pBet-Tac (RFP) Cho=10000; IPTG=500	pBetI input; Cho 10000 µM	Tac input; IPTG 500 µM	Cho 10000 µM + IPTG 500 µM
pBet-Van (GFP) Cho=10000; Van=100	pBetI input; Cho 10000 µM	Van input; Van 100 µM	Cho 10000 µM + Van 100 µM
pBet-Van (RFP) Cho=10000; Van=100	pBetI input; Cho 10000 µM	Van input; Van 100 µM	Cho 10000 µM + Van 100 µM
pPhlF-Tac (GFP) DAPG=10; IPTG=1000	pPhlF input; DAPG 10 µM	Tac input; IPTG 1000 µM	DAPG 10 µM + IPTG 1000 µM
pPhlF-Tac (RFP) DAPG=10; IPTG=1000	pPhlF input; DAPG 10 µM	Tac input; IPTG 1000 µM	DAPG 10 µM + IPTG 1000 µM
pPhlF-Tet (RFP) DAPG=10; aTc=0.2	pPhlF input; DAPG 10 µM	Tet input; aTc 0.2 µM	DAPG 10 µM + aTc 0.2 µM
pTac-Tet (GFP) IPTG=1000; aTc=0.2	pTac input; IPTG 1000 µM	Tet input; aTc 0.2 µM	IPTG 1000 µM + aTc 0.2 µM
pTet-Bet (GFP) aTc=100; Cho=10000	pTet* input; aTc 100 ng mL−1	Bet input; Cho 10000 µM	aTc 100 ng mL−1 + Cho 10000 µM

c

Promoter-reporter and source grid label	[10] first/template input ON	[01] second/added input ON	[11] both inputs ON
pTet-Bet (RFP) aTc=0.2; Cho=10000	pTet* input; aTc 0.2 µM	Bet input; Cho 10000 µM	aTc 0.2 µM + Cho 10000 µM
pTet-PhlF (RFP) aTc=0.2; DAPG=10	pTet* input; aTc 0.2 µM	PhlF input; DAPG 10 µM	aTc 0.2 µM + DAPG 10 µM
pTet-Ttg (GFP) aTc=100; Nar=280	pTet* input; aTc 100 ng mL−1	Ttg input; Nar 280 µM	aTc 100 ng mL−1 + Nar 280 µM
pTet-Ttg (RFP) aTc=100; Nar=280	pTet* input; aTc 100 ng mL−1	Ttg input; Nar 280 µM	aTc 100 ng mL−1 + Nar 280 µM
pTet-Van (GFP) aTc=0.2; Van=100	pTet* input; aTc 0.2 µM	Van input; Van 100 µM	aTc 0.2 µM + Van 100 µM
pTet-Van (RFP) aTc=0.2; Van=100	pTet* input; aTc 0.2 µM	Van input; Van 100 µM	aTc 0.2 µM + Van 100 µM
pTtg-Tac (GFP) Nar=280; IPTG=1000	pTtg input; Nar 280 µM	Tac input; IPTG 1000 µM	Nar 280 µM + IPTG 1000 µM
pTtg-Tac (RFP) Nar=280; IPTG=500	pTtg input; Nar 280 µM	Tac input; IPTG 500 µM	Nar 280 µM + IPTG 500 µM
pVan-Tac (RFP) Van=21; IPTG=1000	pVanCC input; Van 21 µM	Tac input; IPTG 1000 µM	Van 21 µM + IPTG 1000 µM

Promoter-reporter and source grid label	[10] first/template input ON	[01] second/added input ON	[11] both inputs ON
pVan-Ttg (RFP)<br>Van=100; Nar=1000	pVanCC input; Van 100 μM	Ttg input; Nar 1000 μM	Van 100 μM + Nar 1000 μM

Supplementary Table S6 | Selected inducer concentrations and truth-table digit mapping. a, Inducer identity and units. **b,** Selected non-zero working concentrations for the analysed truth-table grids. **c,** Continuation of selected non-zero working concentrations for the analysed truth-table grids. Concentrations are the selected non-zero working concentrations for the analysed four-state grid, not stock concentrations. The source grid label is preserved exactly as used in Supplementary Table S2 and the source workbooks. State [00] contains neither inducer, [10] contains only the first/template-input inducer, [01] contains only the second/added-input inducer, and [11] contains both. For aTc, the source workbooks use two notations: rows labelled aTc=0.2 are reported as 0.2 μM, whereas rows labelled aTc=100 are reported as 100 ng mL−1; this table keeps those source-specific unit conventions explicit.

Promoter Name	Template Region (-35/-10)	Added Operator Position	Full Sequence (5' to 3')
pTac-Van	**pTac template**: -37 to +19; -35: -35 to -30; -10: -13 to -8; +1: +1<br>Underlined overlap: **lacOsym** -1 to +19 within pTac template; underlined bases use the larger template-feature colour.	**VanO2**: +20 to +49	tgttgacaattaatcatcggctcgtataatgtgtggaattgtgagcgctcacaattATTGGATCCAATagcgctATTGGATCCAAT
pTet-Bet	**pTet* template**: -76 to -1; -35: -35 to -30; -10: -12 to -7<br>Underlined overlap: **tetO2** -54 to -36 within pTet* template; underlined bases use the larger template-feature colour.<br>Underlined overlap: **tetO2** -29 to -11 within pTet* template; underlined bases use the larger template-feature colour.	**BetO**: +1 to +22	ttttcagcaggacgcactgacctccctatcagtgatagagattgacatccctatcagtgatagagatactgagcacttatattgaacgtccaatcaat
pTet-PhlF	**pTet* template**: -76 to -1; -35: -35 to -30; -10: -12 to -7<br>Underlined overlap: **tetO2** -54 to -36 within pTet* template; underlined bases use the larger template-feature colour.<br>Underlined overlap: **tetO2** -29 to -11 within pTet* template; underlined bases use the larger template-feature colour.	**PhlO**: +1 to +30	ttttcagcaggacgcactgacctccctatcagtgatagagattgacatccctatcagtgatagagatactgagcacaccttaacgatacggtacgtttcgtatcat
pTet-Ttg	**pTet* template**: -76 to -1; -35: -35 to -30; -10: -12 to -7<br>Underlined overlap: **tetO2** -54 to -36 within pTet* template; underlined bases use the larger template-feature colour.<br>Underlined overlap: **tetO2** -29 to -11 within pTet* template; underlined bases use the larger template-feature colour.	**TtgO**: +1 to +30	ttttcagcaggacgcactgacctccctatcagtgatagagattgacatccctatcagtgatagagatactgagcacATACTTACATTCATGGTTGTTTGTAAATAC
pTet-Van	**pTet* template**: -76 to -1; -35: -35 to -30; -10: -12 to -7<br>Underlined overlap: **tetO2** -54 to -36 within pTet* template; underlined bases use the larger template-feature colour.<br>Underlined overlap: **tetO2** -29 to -11 within pTet* template; underlined bases use the larger template-feature colour.	**VanO2**: +1 to +30	ttttcagcaggacgcactgacctccctatcagtgatagagattgacatccctatcagtgatagagatactgagcacATTGGATCCAATagcgctATTGGATCCAAT
pBet-Tac	**pBetI template**: -63 to -1; -35: -35 to -30; -10: -12 to -7<br>Underlined overlap: **BetO** -29 to -8 within pBetI template; underlined bases use the larger template-feature colour.	**lacOsym**: +1 to +20	agcgcgggtgagagggattcgttaccaatagacaattgattggacgttcaatataatgctagcaattgtgagcgctcacaatt
pPhlF-Tac	**pPhlF template**: -66 to -1; -35: -35 to -31; -10: -12 to -7<br>Underlined overlap: **native PhlO** -30 to -1 within pPhlF template; underlined bases use the larger template-feature colour.	**lacOsym**: +1 to +20	cgacgtacggtggaatctgattcgttaccaattgacatgatacgaaacgtaccgtatcgttaaggtaattgtgagcgctcacaatt
pTtg-Tac	**pTtg template**: -48 to +3; -35: -36 to -31; -10: -10 to -1; +1: +1<br>Underlined overlap: **native TtgO** -30 to -11 within pTtg template; underlined bases use the larger template-feature colour.	**lacOsym**: +4 to +23	cacccagcagtatttacaaacaaccatgaatgtaagtatattccttagcaaaattgtgagcgctcacaatt

pVan-Tac	**pVanCC template**: -46 to +4; -35: -35 to -30; -10: -12 to -7; +1: +1<br>Underlined overlap: **VanO2 half-site** -46 to -36 within pVanCC template; underlined bases use the larger template-feature colour.<br>Underlined overlap: **VanO2 half-site** -8 to +3 within pVanCC template; underlined bases use the larger template-feature colour.	**lacOsym**: +5 to +24	attggatccaattgacagctagctcagtcctaggtaccattggatccaataattgtgagcgctcacaatt
pVan-Ttg	**pVanCC template**: -46 to +4; -35: -35 to -30; -10: -12 to -7; +1: +1<br>Underlined overlap: **VanO2 half-site** -46 to -36 within pVanCC template; underlined bases use the larger template-feature colour.<br>Underlined overlap: **VanO2 half-site** -8 to +3 within pVanCC template; underlined bases use the larger template-feature colour.	**TtgO**: +5 to +34	attggatccaattgacagctagctcagtcctaggtaccattggatccaatATACTTACATTCATGGTTGTTTGTAAATAC
pTac-Tet	**pTac template**: -37 to +19; -35: -35 to -30; -10: -13 to -8; +1: +1<br>Underlined overlap: **lacOsym** -1 to +19 within pTac template; underlined bases use the larger template-feature colour.	**tetO2**: +20 to +38; **lacO (native)**: +39 to +55	tgttgacaattaatcatcggctcgtataatgtgtggaattgtgagcgctcacaatttccctatcagtgatagagaTTGTGAGCGGATAACAA
pBet-Van	**pBetI template**: -63 to -1; -35: -35 to -30; -10: -12 to -7<br>Underlined overlap: **BetO** -29 to -8 within pBetI template; underlined bases use the larger template-feature colour.	**VanO2**: +1 to +30	agcgcgggtgagagggattcgttaccaatagacaattgattggacgttcaatataatgctagcATTGGATCCAATagcgctATTGGATCCAAT
pPhlF-Tet	**pPhlF template**: -66 to -1; -35: -35 to -31; -10: -12 to -7<br>Underlined overlap: **native PhlO** -30 to -1 within pPhlF template; underlined bases use the larger template-feature colour.	**tetO2**: +1 to +19	cgacgtacggtggaatctgattcgttaccaattgacatgatacgaaacgtaccgtatcgttaaggttccctatcagtgatagaga

Supplementary Table S7 | Compact promoter sequence annotations for engineered promoter architectures. This consolidated table reports promoter name, annotated template -35/-10 region, added-operator position relative to +1, and full 5' to 3' sequence.

Promoter	Reporter	Main figures	Supplementary figures
pBet-Tac	GFP	Fig. 1b; Fig. 2; Fig. 3	Fig. S13a
pBet-Tac	RFP	Fig. 1b; Fig. 2; Fig. 3; Fig. 4; Fig. 5	Fig. S13b; Fig. S2
pBet-Van	GFP	Fig. 1b; Fig. 2; Fig. 3; Fig. 4; Fig. 5	Fig. S13c; Fig. S3
pBet-Van	RFP	Fig. 1b; Fig. 2; Fig. 3	Fig. S13d
pPhlF-Tac	GFP	Fig. 1b; Fig. 2; Fig. 3	Fig. S14a
pPhlF-Tac	RFP	Fig. 1b; Fig. 2; Fig. 3; Fig. 4; Fig. 5	Fig. S14b; Fig. S4
pPhlF-Tet	RFP	Fig. 1b; Fig. 2; Fig. 3; Fig. 4; Fig. 5	Fig. S14c; Fig. S5
pTac-Tet	GFP	Fig. 1b; Fig. 2; Fig. 3; Fig. 5	Fig. S14d; Fig. S6
pTet-Bet	GFP	Fig. 1b; Fig. 2; Fig. 3	Fig. S15a
pTet-Bet	RFP	Fig. 1b; Fig. 2; Fig. 3; Fig. 4; Fig. 5	Fig. S15b; Fig. S7
pTet-PhlF	RFP	Fig. 1b; Fig. 2; Fig. 3; Fig. 4; Fig. 5	Fig. S15c; Fig. S1
pTet-Ttg	GFP	Fig. 1b; Fig. 2; Fig. 3	Fig. S15d

pTet-Ttg	RFP	Fig. 1b; Fig. 2; Fig. 3; Fig. 4; Fig. 5	Fig. S16a; Fig. S8
pTet-Van	GFP	Fig. 1b; Fig. 2; Fig. 3	Fig. S16b
pTet-Van	RFP	Fig. 1b; Fig. 2; Fig. 3; Fig. 4; Fig. 5	Fig. S16c; Fig. S9
pTtg-Tac	GFP	Fig. 1b; Fig. 2; Fig. 3	Fig. S16d
pTtg-Tac	RFP	Fig. 1b; Fig. 2; Fig. 3; Fig. 4; Fig. 5	Fig. S16e; Fig. S10
pVan-Tac	RFP	Fig. 1b; Fig. 2; Fig. 3; Fig. 5	Fig. S16f; Fig. S11
pVan-Ttg	RFP	Fig. 1b; Fig. 2; Fig. 3; Fig. 5	Fig. S16g; Fig. S12
pTac-Van	GFP	Fig. 5	Fig. S17
pTac-Van	RFP	not shown	Fig. S17

Supplementary Table S8 | Cross-reference between characterised promoter/reporter constructs and the current manuscript and supplementary figure panels. Supplementary Table S2 contains all 19 curated promoter-reporter truth tables; Supplementary Table S12 contains the template-promoter conditional activation-fold control analysis.

```
cggccttttacggttcctggccttttgctggccttttgctcacatgttctttcctgcgttatcccctgattctgtggat
aaccgtattaccgcctttgagtgagctgataccgctcgccgcagccgaacgaccgagcgcagcgagtaagaggacatccg
gtcaaataaaacgaaaggctcagtcgaaagactgggcctttcgttttagacTTCAGCCAAAAAACTTAAGACCGCCGGTC
TTGTCCACTACCTTGCAGTAATGCGGTGGACAGGATCGGCGGTTTTCTTTTCTCTTCTCAAAATGCCaGAGACCcggaaG
GCGCGCCaagattGGTCTCaCCGCAAATCGCGGCCTTTTTTATTGATAACAAAAAGGCGCTACTACTAAACGCCAGAGTa
gcgcgggtgagagggattcgttaccaatagacaattgattggacgttcaatataatgctagcaattgtgagcgctcacaa
ttAAGATCGGGTGTAAACagctgtcaccggatgtgctttccggtctgatgagtccgtgaggacgaaacagcctctacaaa
taattttgtttaaTgatAGAGAAAGAGGGGAAAgtaTAGatggtgagcaagggcgaggaggataacatggccatcatcaa
ggagttcatgcgcttcaaggtgcacatggagggctccgtgaacggccacgagttcgagatcgagggcgagggcgagggcc
gcccctacgagggcacccagaccgccaagctgaaggtgaccaagggtggccccctgcccttcgcctgggacatcctgtcc
cctcagttcatgtacggctccaaggcctacgtgaagcaccccgccgacatccccgactacttgaagctgtccttccccga
gggcttcaagtgggagcgcgtgatgaacttcgaggacggcggcgtggtgaccgtgacccaggactcctccttgcaggacg
gcgagttcatctacaaggtgaagctgcgcggcaccaacttcccctccgacggccccgtaatgcagaagaagaccatgggc
tgggaggcctcctccgagcggatgtaccccgaggacggcgccctgaagggcgagatcaagcagaggctgaagctgaagga
cggcggccactacgacgctgaggtcaagaccacctacaaggccaagaagcccgtgcagctgcccggcgcctacaacgtca
acatcaagttggacatcacctcccacaacgaggactacaccatcgtggaacagtacgaacgcgccgagggccgccactcc
accggcggcatggacgagctgtacaagGGCTCGGGATAATAACCTAGAGAAAGAGGGGAAATACTAGATGgagaaaaaaa
tcactggatataccaccgttgatatatcccaatggcatcgtaaagaacattttgaggcatttcagtcagttgctcaatgt
acctataaccagaccgttcagctggatattacggcctttttaaagaccgtaaagaaaaataagcacaagttttatccggc
ctttattcacattcttgcccgcctgatgaatgctcatccggagttccgtatggcaatgaaagacggtgagctggtgatat
gggatagtgttcacccttgttacaccgttttccatgagcaaactgaaacgttttcatcgctctggagtgaataccacgac
gatttccggcagtttctacacatatattcgcaagatgtggcgtgttacggtgaaaacctggcctatttccctaaagggtt
tattgagaatatgtttttcgtctcagccaatccctgggtgagtttcaccagttttgatttaaacgtggccaatatggaca
acttcttcgcccccgttttcactatgggcaaatattatacgcaaggcgacaaggtgctgatgccgctggcgattcaggtt
catcatgccgtttgtgatggcttccatgtcggcagaatgcttaatgaattacaacagtactgcgatgagtggcagggcgg
ggcgggcagcggttctggctccagccatattcaacgggaaacgtcttgctcgaggccgcgattaaattccaacatggatg
ctgatttatatgggtataaatgggctcgcgataatgtcgggcaatcaggtgcgacaatctatcgattgtatgggaagccc
gatgcgccagagttgtttctgaaacatggcaaaggtagcgttgccaatgatgttacagatgagatggtcagactaaactg
gctgacggaatttatgcctcttccgaccatcaagcattttatccgtactcctgatgatgcatggttactcaccactgcga
tccccgggaaaacagcattccaggtattagaagaatatcctgattcaggtgaaaatattgttgatgcgctggcagtgttc
ctgcgccggttgcattcgattcctgtttgtaattgtccttttaacagcgatcgcgtatttcgtctcgctcaggcgcaatc
acgaatgaataacggtttggttgatgcgagtgattttgatgacgagcgtaatggctggcctgttgaacaagtctggaaag
aaatgcataagcttttgccattctcaccggattcagtcgtcactcatggtgatttctcacttgataaccttatttttgac
gaggggaaattaataggttgtattgctgttggacgagtcggaatcgcagaccgataccaggatcttgccatcctatggaa
ctgcctcggtgagttttctccttcattacagaaacggctttttcaaaaatatggtattgataatcctgatatgaataaat
tgcagtttcatttgatgctcgatgagtttttctaactgtcagaccaagtttactcatatatactttagattgatttaaaa
cttcatttttaatttcttggactcctgttgatagatccagtaatgacctcagaactccatctggatttgttcagaacgct
cggttgccgccgggcgtttttttattggtgagaatccaggggtccccaataattacgatttaaattagtagcccgcctaat
gagcgggcttttttttaattcccctatttgtttatttttctaaatacattcaaatatgtatccgctcatgagacaataac
cctgataaatgcttcaataatattgaaaaaggaagagtatgagcattcagcattttcgtgtggcgctgattccgtttttt
gcggcgttttgcctgccggtgtttgcgcatccggaaaccctggtgaaagtgaaagatgcggaagatcaactgggtgcgcg
cgtgggctatattgaactggatctgaacagcggcaaaattctggaatcttttcgtccggaagaacgttttccgatgatga
gcacctttaaagtgctgctgtgcggtgcggttctgagccgtgtggatgcgggccaggaacaactgggccgtcgtattcat
tatagccagaacgatctggtggaatatagcccggtgaccgaaaaacatctgaccgatggcatgaccgtgcgtgaactgtg
cagcgcggcgattaccatgagcgataacaccgcggcgaacctgctgctgacgaccattggcggtccgaaagaactgaccg
cgtttctgcataacatgggcgatcatgtgacccgtctggatcgttgggaaccggaactgaacgaagcgattccgaacgat
gaacgtgataccaccatgccggcagcaatggcgaccaccctgcgtaaactgctgacgggtgagctgctgaccctggcaag
ccgccagcaactgattgattggatggaagcggataaagtggcgggtccgctgctgcgtagcgcgctgccggctggctggt
ttattgcggataaaagcggtgcgggcgaacgtggcagccgtggcattattgcggcgctgggcccggatggtaaaccgagc
cgtattgtggtgatttataccaccggcagccaggcgacgatggatgaacgtaaccgtcagattgcggaaattggcgcgag
cctgattaaacattggtaaaccgatacaattaaaggctcctttttggagcctttttttttggaaaaaggatctaggtgaag
atcctttttgataatctcatgaccaaaatcccttaacgtgagttttcgttccactgagcgtcagaccccgtagaaaagat
caaaggatcttcttgagatcctttttttCctgcgcgtaatctgctgcttgcaaacaaaaaaaccaccgctaccagcggtgg
tttgtttgccggatcaagagctaccaactctttttccgaaggtaactggcttcagcagagcgcagataccaaatactgtC
cttctagtgtagccgtagttaggccaccacttcaagaactctgtagcaccgcctacatacctcgctctgctaatcctgtt
accagtggctgctgccagtggcgataagtcgtgtcttaccgggttggactcaagacgatagttaccggataaggcgcagc
ggtcgggctgaacggggggttcgtgcacacagcccagcttggagcgaacgacctacaccgaactgagatacctacagcgt
gagctatgagaaagcgccacgcttcccgaagggagaaaggcggacaggtatccggtaagcggcagggtcggaacaggaga
gcgcacgagggagcttccagggggaaacgcctggtatctttatagtcctgtcgggtttcgccacctctgacttgagcgtc
gatttttgtgatgctcgtcaggggggcggagcctatggaaaaacgccagcaacg
```

Supplementary Table S9 | Representative mCherry plasmid sequence. The complete plasmid sequence is shown as a single continuous entry, with one consistent colour assigned to each annotated feature across all of its segments. Feature key: rrnB T1 terminator; spy strong terminator; 18a-AAN-ONE gBlock; L3S1P22 strong terminator; gBlock 8xgRNA; pBetI promoter (Cho); lac operator (symmetric); RiboJ ribozyme insulator; RBS-64; mCherry; gBlock AAN-mCherry-R4-TWO-B; cat (CmR) CDS chloramphenicol resistance protein; 3GS linker; dKanR; TAMPC cassette from SEVA191; lambda t0 terminator; AmpR promoter; AmpR; fd terminator; ColE1 promoter; RNAII ColE1; ColE1 P1 RNA I promoter; pMB1 mutated to medium copy number.

```
cggcctttttacggttcctggccttttgctggccttttgctcacatgttctttcctgcgttatcccctgattctgtgg
ataaccgtattaccgcctttgagtgagctgataccgctcgccgcagccgaacgaccgagcgcagcgagtaagaggaca
tccggtcaaataaaacgaaaggctcagtcgaaagactgggcctttcgttttagacTTCAGCCAAAAAACTTAAGACCG
CCGGTCTTGTCCACTACCTTGCAGTAATGCGGTGGACAGGATCGGCGGTTTTCTTTTCTCTTCTCAAAATGCCaGAGA
CCcggaaGGCGCGCCaagattGGTCTCaCCGCAAATCGCGGCCTTTTTTATTGATAACAAAAAGGCGCTACTACTAAA
CGCCAGAGTagcgcgggtgagagggattcgttaccaatagacaattgattggacgttcaatataatgctagcaattgt
gagcgctcacaattAAGATCGGGTGTAAACagctgtcaccggatgtgctttccggtctgatgagtccgtgaggacgaa
acagcctctacaaataatttgtttaaTgatAGAGAAAGAGGGGAAAgtaTAGatggtgagcaagggcgaggagctgt
tcaccggggtggtgcccatcctggtcgagctggacggcgacgtaaacggccacaagttcagcgtgtccggcgagggcg
agggcgatgccacctacggcaagctgaccctgaagttcatctgcaccaccggcaagctgcccgtgccctggcccaccc
tcgtgaccaccctgacctacggcgtgcagtgcttcagccgctaccccgaccacatgaagcagcacgacttcttcaagt
ccgccatgcccgaaggctacgtccaggagcgcaccatcttcttcaaggacgacggcaactacaagacccgcgccgagg
tgaagttcgagggcgacaccctggtgaaccgcatcgagctgaagggcatcgacttcaaggaggacggcaacatcctgg
ggcacaagctggagtacaactacaacagccacaacgtctatatcatggccgacaagcagaagaacggcatcaaggtga
acttcaagatccgccacaacatcgaggacggcagcgtgcagctcgccgaccactaccagcagaacacccccatcggcg
acggccccgtgctgctgcccgacaaccactacctgagcacccagtccgccctgagcaaagaccccaacgagaagcgcg
atcacatggtcctActggagttcgtgaccgccgccgggatcactctcggcatggacgagctgtacaagTAATAACCTA
GAGAAAGAGGGGAAATACTAGATGgagaaaaaaatcactggatataccaccgttgatatatcccaatggcatcgtaaa
gaacattttgaggcatttcagtcagttactcaatgtacctataaccagaccgttcagctggatattacggcctttta
aagaccgtaaagaaaaataagcacaagttttatccggcctttattcacattcttgcccgcctgatgaatgctcatccg
gagttccgtatggcaatgaaagacggtgagctggtgatatgggatagtgttcacccttgttacaccgttttccatgag
caaactgaaacgttttcatcgctctggagtgaataccacgacgatttccggcagtttctacacatatattcgcaagat
gtggcgtgttacggtgaaaacctggcctatttccctaaagggtttattgagaatatgtttttcgtctcagccaatccc
tgggtgagtttcaccagttttgatttaaacgtggccaatatggacaacttcttcgcccccgttttcactatgggcaaa
tattatacgcaaggcgacaaggtgctgatgccgctggcgattcaggttcatgctgccgtttgtgatggcttccatgtc
ggcagaatgcttaatgaattacaacagtactgcgatgagtggcagggcggggcgggcagcggttctggctccagccat
attcaacgggaaacgtcttgctctaggccgcgattaaattccaacatggatgctgatttatatgggtataaatgggct
cgcgataatgtcgggcaatcaggtgcgacaatctatcgattgtatgggaagcccgatgcgccagagttgtttctgaaa
catggcaaaggtagcgttgccaatgatgttacagatgagatggtcagactaaactggctgacggaatttatgcctctt
ccgaccatcaagcattttatccgtactcctgatgatgcatggttactcaccactgcgatccccgggaaaacagcattc
caggtattagaagaatatcctgattcaggtgaaaatattgttgatgcgctggcagtgttcctgcgccggttgcattcg
attcctgtttgtaattgtccttttaacagcgaccgcgtatttcgtctcgctcaggcgcaatcacgaatgaataacggt
ttggttgatgcgagtgattttgatgacgagcgtaatggctggcctgttgaacaagtctggaaagaaatgcataaactt
ttgccattctcaccggattcagtcgtcactcatggtgatttctcacttgataaccttatttttgacgaggggaaatta
ataggttgtattgatgttggacgagtcggaatcgcagaccgataccaggatcttgccatcctatggaactgcctcggt
gagttttctccttcattacagaaacggctttttcaaaaatatggtattgataatcctgatatgaataaattgcagttt
catttgatgctcgatgagtttttctaactgtcagaccaagtttactcatatatactttagattgatttaaaacttcat
ttttaatttcttggactcctgttgatagatccagtaatgacctcagaactccatctggatttgttcagaacgctcggt
tgccgccgggcgttttttattggtgagaatccaggggtccccaataattacgatttaaattagtagcccgcctaatga
gcgggcttttttttaattcccctatttgtttatttttctaaatacattcaaatatgtatccgctcatgagacaataac
cctgataaatgcttcaataatattgaaaaaggaagagtatgagcattcagcattttcgtgtggcgctgattccgtttt
ttgcggcgttttgcctgccggtgtttgcgcatccggaaaccctggtgaaagtgaaagatgcggaagatcaactgggtg
cgcgcgtgggctatattgaactggatctgaacagcggcaaaattctggaatcttttcgtccggaagaacgttttccga
tgatgagcacctttaaagtgctgctgtgcggtgcggttctgagccgtgtggatgcgggccaggaacaactgggccgtc
gtattcattatagccagaacgatctggtggaatatagcccggtgaccgaaaaacatctgaccgatggcatgaccgtgc
gtgaactgtgcagcgcggcgattaccatgagcgataacaccgcggcgaacctgctgctgacgaccattggcggtccga
aagaactgaccgcgtttctgcataacatgggcgatcatgtgacccgtctggatcgttgggaaccggaactgaacgaag
cgattccgaacgatgaacgtgataccaccatgccggcagcaatggcgaccaccctgcgtaaactgctgacgggtgagc
tgctgaccctggcaagccgccagcaactgattgattggatggaagcggataaagtggcgggtccgctgctgcgtagcg
cgctgccggctggctggtttattgcggataaaagcggtgcgggcgaacgtggcagccgtggcattattgcggcgctgg
gcccggatggtaaaccgagccgtattgtggtgatttataccaccggcagccaggcgacgatggatgaacgtaaccgtc
agattgcggaaattggcgcgagcctgattaaacattggtaaaccgatacaattaaaggctccttttggagcctttttt
tttggaaaaaggatctaggtgaagatcctttttgataatctcatgaccaaaatcccttaacgtgagttttcgttccac
tgagcgtcagaccccgtagaaaagatcaaaggatcttcttgagatcctttttttCctgcgcgtaatctgctgcttgcaa
acaaaaaaaccaccgctaccagcggtggtttgtttgccggatcaagagctaccaactctttttccgaaggtaactggc
ttcagcagagcgcagataccaaatactgtCcttctagtgtagccgtagttaggccaccacttcaagaactctgtagca
ccgcctacatacctcgctctgctaatcctgttaccagtggctgctgccagtggcgataagtcgtgtcttaccgggttg
gactcaagacgatagttaccggataaggcgcagcggtcgggctgaacggggggttcgtgcacacagcccagcttggag
cgaacgacctacaccgaactgagatacctacagcgtgagctatgagaaagcgccacgcttcccgaagggagaaaggcg
gacaggtatccggtaagcggcagggtcggaacaggagagcgcacgagggagcttccagggggaaacgcctggtatctt
tatagtcctgtcgggtttcgccacctctgacttgagcgtcgatttttgtgatgctcgtcaggggggcggagcctatgg
aaaaacgccagcaacg
```

Supplementary Table S10 | Representative EGFP plasmid sequence. The complete plasmid sequence is shown as a single continuous entry, with one consistent colour assigned to each annotated feature across all of its segments. Feature key: rrnB T1 terminator; spy strong terminator; 18a-AAN-ONE gBlock; L3S1P22 strong terminator; gBlock 8xgRNA; pBetI promoter (Cho); lac operator (symmetric); RiboJ ribozyme insulator; RBS-64; EGFP; dCmR (chloramphenicol resistance knockout); 3GS linker; KanR; TAMPC cassette from SEVA191; lambda t0 terminator; AmpR promoter; AmpR; fd terminator; ColE1 promoter; RNAII ColE1; ColE1 P1 RNA I promoter; pMB1 mutated to medium copy number.

Architecture	Template	Added input	Added operator	Orientation	Approximate added-site position	Role	Selected reporter	Selected call
pTet-PhlF	pTet*	PhlF	PhlO	Reverse	Downstream (+1 to +30)	Curated	RFP	Pass
pBet-Tac	pBetI	Tac	lacOsym	Forward	Downstream (+1 to +20)	Curated	RFP	Pass
pBet-Van	pBetI	Van	VanO2	Forward	Downstream (+1 to +30)	Curated	GFP	Pass
pPhlF-Tac	pPhlF	Tac	lacOsym	Forward	Downstream (+1 to +20)	Curated	RFP	Pass
pPhlF-Tet	pPhlF	Tet	tetO2	Forward	Downstream (+1 to +19)	Curated	RFP	Pass
pTac-Tet	pTac	Tet	tetO2	Forward	Distal downstream (+20 to +38)	Curated	GFP	Fail
pTet-Bet	pTet*	Bet	BetO	Forward	Downstream (+1 to +22)	Curated	RFP	Pass
pTet-Ttg	pTet*	Ttg	TtgO	Reverse	Downstream (+1 to +30)	Curated	RFP	Pass
pTet-Van	pTet*	Van	VanO2	Forward	Downstream (+1 to +30)	Curated	RFP	Pass
pTtg-Tac	pTtg	Tac	lacOsym	Forward	Downstream (+4 to +23)	Curated	RFP	Pass
pVan-Tac	pVanCC	Tac	lacOsym	Forward	Downstream (+5 to +24)	Curated	RFP	N.C.
pVan-Ttg	pVanCC	Ttg	TtgO	Reverse	Downstream (+5 to +34)	Curated	RFP	Fail
pTac-Van	pTac	Van	VanO2	Forward	Exploratory downstream insertion	Exploratory	Incomplete non-toxic truth table	Not included in curated comparison

Supplementary Table S11 | Overview of the engineered promoter architectures. This table consolidates the scaffold, added operator, orientation, approximate downstream position, curated versus exploratory status, and selected reporter call for the promoter architectures discussed across Supplementary Table S7.

a

Template	AND promoter	Role	Template inducer	Added inducer	AND reporter	Template reporter	Requested conc.	Used conc.	Match	Distance
pBetI	pBet-Tac	curated	Cho	IPTG	GFP	GFP	10000	10000	exact	0.000
pBetI	pBet-Tac	curated	Cho	IPTG	RFP	GFP	10000	10000	exact	0.000
pBetI	pBet-Van	curated	Cho	Van	GFP	GFP	10000	10000	exact	0.000
pBetI	pBet-Van	curated	Cho	Van	RFP	GFP	10000	10000	exact	0.000
pPhlF	pPhlF-Tac	curated	DAPG	IPTG	GFP	GFP	10.00	10.00	exact	0.000
pPhlF	pPhlF-Tac	curated	DAPG	IPTG	RFP	RFP	10.00	2.100	nearest_available	7.900
pPhlF	pPhlF-Tet	curated	DAPG	aTc	RFP	RFP	10.00	2.100	nearest_available	7.900
pTac	pTac-Tet	curated	IPTG	aTc	GFP	GFP	1000	500.00	nearest_available	500.00
pTet*	pTet-Bet	curated	aTc	Cho	GFP	GFP	100.00	100.00	exact	0.000
pTet*	pTet-Bet	curated	aTc	Cho	RFP	RFP	0.200	0.200	exact	0.000
pTet*	pTet-PhlF	curated	aTc	DAPG	RFP	RFP	0.200	0.200	exact	0.000
pTet*	pTet-Ttg	curated	aTc	Nar	GFP	GFP	100.00	100.00	exact	0.000
pTet*	pTet-Ttg	curated	aTc	Nar	RFP	RFP	100.00	0.200	nearest_available	99.80
pTet*	pTet-Van	curated	aTc	Van	GFP	GFP	0.200	0.200	exact	0.000
pTet*	pTet-Van	curated	aTc	Van	RFP	RFP	0.200	0.200	exact	0.000
pTtg	pTtg-Tac	curated	Nar	IPTG	GFP	GFP	280.00	280.00	exact	0.000
pTtg	pTtg-Tac	curated	Nar	IPTG	RFP	RFP	280.00	280.00	exact	0.000
pVanCC	pVan-Tac	curated	Van	IPTG	RFP	RFP	21.00	100.00	nearest_available	79.00
pVanCC	pVan-Ttg	curated	Van	Nar	RFP	RFP	100.00	100.00	exact	0.000
pTac	pTac-Van	exploratory	IPTG	Van	GFP	GFP	1000	500.00	nearest_available	500.00
pTac	pTac-Van	exploratory	IPTG	Van	RFP	RFP	1000	500.00	nearest_available	500.00

b

Template	AND promoter	Reporter	Template [0] mean	Template [1] mean	Template [0] sd	Template [1] sd	n [0]	n [1]	R_YES	log2 R_YES	CI low	CI high
pBetI	pBet-Tac	GFP	7396	49143	0.000	4601	1	8	6.644	2.732	2.648	2.820
pBetI	pBet-Tac	RFP	7396	49143	0.000	4601	1	8	6.644	2.732	2.645	2.821
pBetI	pBet-Van	GFP	7396	49143	0.000	4601	1	8	6.644	2.732	2.649	2.820
pBetI	pBet-Van	RFP	7396	49143	0.000	4601	1	8	6.644	2.732	2.648	2.820
pPhlF	pPhlF-Tac	GFP	62615	64180	2145	282.10	4	2	1.025	0.036	-0.006	0.080
pPhlF	pPhlF-Tac	RFP	13678	54997	276.57	4023	3	3	4.021	2.008	1.900	2.106
pPhlF	pPhlF-Tet	RFP	13678	54997	276.57	4023	3	3	4.021	2.008	1.900	2.106
pTac	pTac-Tet	GFP	6611	76500	345.73	2528	6	6	11.57	3.533	3.467	3.598
pTet*	pTet-Bet	GFP	6578	70382	153.50	0.000	3	1	10.70	3.420	3.394	3.458
pTet*	pTet-Bet	RFP	0.000	34173	0.000	5038	3	12	1667	10.70	10.58	10.81
pTet*	pTet-PhlF	RFP	0.000	34173	0.000	5038	3	12	1667	10.70	10.58	10.81

pTet*	pTet-Ttg	GFP	6578	70382	153.50	0.000	3	1	10.70	3.420	3.394	3.458
pTet*	pTet-Ttg	RFP	0.000	34173	0.000	5038	3	12	1667	10.70	10.58	10.81
pTet*	pTet-Van	GFP	6578	56517	153.50	3215	3	18	8.592	3.103	3.055	3.152
pTet*	pTet-Van	RFP	0.000	34173	0.000	5038	3	12	1667	10.70	10.58	10.81
pTtg	pTtg-Tac	GFP	8463	43899	666.68	2782	3	13	5.187	2.375	2.267	2.494
pTtg	pTtg-Tac	RFP	31.24	7322	0.000	338.84	1	12	234.39	7.873	7.835	7.908
pVanCC	pVan-Tac	RFP	0.000	55931	0.000	4339	6	7	2729	11.41	11.34	11.49
pVanCC	pVan-Ttg	RFP	0.000	55931	0.000	4339	6	7	2729	11.41	11.34	11.49
pTac	pTac-Van	GFP	6611	76500	345.73	2528	6	6	11.57	3.533	3.468	3.597
pTac	pTac-Van	RFP	0.000	32917	0.000	25551	3	17	1606	10.65	9.975	11.08

c

Template	AND promoter	Reporter	AND [01] mean	AND [11] mean	AND [01] sd	AND [11] sd	n [01]	n [11]	R_AND	log2 R_AND	CI low	CI high
pBetI	pBet-Tac	GFP	6206	46516	141.36	1651	3	3	7.495	2.906	2.851	2.960
pBetI	pBet-Tac	RFP	0.000	6139	0.000	143.08	3	3	299.55	8.227	8.193	8.260
pBetI	pBet-Van	GFP	5741	59190	217.03	1099	3	3	10.31	3.366	3.313	3.420
pBetI	pBet-Van	RFP	0.000	0.000	0.000	0.000	3	3	0.000			
pPhlF	pPhlF-Tac	GFP	61486	71953	2243	678.67	3	3	1.170	0.227	0.171	0.268
pPhlF	pPhlF-Tac	RFP	2988	13564	228.75	258.88	3	3	4.539	2.182	2.083	2.294
pPhlF	pPhlF-Tet	RFP	16800	63107	1509	1653	3	3	3.756	1.909	1.797	2.047
pTac	pTac-Tet	GFP	38428	74064	26605	1406	3	3	1.927	0.947	0.440	3.255
pTet*	pTet-Bet	GFP	6455	26924	761.93	1297	2	2	4.171	2.060	1.895	2.234
pTet*	pTet-Bet	RFP	0.000	8479	0.000	204.60	3	3	413.74	8.693	8.656	8.725
pTet*	pTet-PhlF	RFP	0.000	38690	0.000	1190	3	3	1888	10.88	10.84	10.92
pTet*	pTet-Ttg	GFP	5806	7813	74.51	679.44	3	6	1.346	0.428	0.335	0.518
pTet*	pTet-Ttg	RFP	0.000	1740	0.000	94.78	3	6	84.88	6.407	6.347	6.460
pTet*	pTet-Van	GFP	6395	8216	99.68	785.88	3	3	1.285	0.361	0.264	0.509
pTet*	pTet-Van	RFP	0.000	28030	0.000	2023	3	3	1368	10.42	10.35	10.53
pTtg	pTtg-Tac	GFP	6389	10002	129.11	754.13	3	3	1.566	0.647	0.563	0.763
pTtg	pTtg-Tac	RFP	0.000	739.87	0.000	15.17	3	3	36.10	5.174	5.145	5.204
pVanCC	pVan-Tac	RFP	0.000	10256	0.000	0.000	4	1	500.44	8.967	8.967	8.967
pVanCC	pVan-Ttg	RFP	0.000	36976	0.000	236.54	3	3	1804	10.82	10.81	10.83
pTac	pTac-Van	GFP	4907	7140	243.82	902.17	3	3	1.455	0.541	0.377	0.719
pTac	pTac-Van	RFP	0.000	37664	0.000	1410	3	3	1838	10.84	10.79	10.89

d

Template	AND promoter	Reporter	Delta log2	Delta CI low	Delta CI high	Ratio of ratios	Denominator nonpositive	Lower bound	Response floor
pBetI	pBet-Tac	GFP	0.174	0.069	0.277	1.128	No	No	
pBetI	pBet-Tac	RFP	5.495	5.401	5.587	45.08	Yes	Yes	20.49
pBetI	pBet-Van	GFP	0.634	0.529	0.734	1.552	No	No	

pBetI	pBet-Van	RFP					Yes	Yes	20.49
pPhlF	pPhlF-Tac	GFP	0.191	0.124	0.255	1.142	No	No	
pPhlF	pPhlF-Tac	RFP	0.175	0.036	0.325	1.129	No	No	
pPhlF	pPhlF-Tet	RFP	-0.098	-0.253	0.068	0.934	No	No	
pTac	pTac-Tet	GFP	-2.586	-3.121	-0.295	0.167	No	No	
pTet*	pTet-Bet	GFP	-1.359	-1.525	-1.185	0.390	No	No	
pTet*	pTet-Bet	RFP	-2.011	-2.121	-1.883	0.248	Yes	Yes	20.49
pTet*	pTet-PhlF	RFP	0.179	0.063	0.307	1.132	Yes	Yes	20.49
pTet*	pTet-Ttg	GFP	-2.991	-3.091	-2.896	0.126	No	No	
pTet*	pTet-Ttg	RFP	-4.296	-4.420	-4.161	0.051	Yes	Yes	20.49
pTet*	pTet-Van	GFP	-2.742	-2.861	-2.598	0.150	No	No	
pTet*	pTet-Van	RFP	-0.286	-0.427	-0.127	0.820	Yes	Yes	20.49
pTtg	pTtg-Tac	GFP	-1.728	-1.886	-1.575	0.302	No	No	
pTtg	pTtg-Tac	RFP	-2.699	-2.743	-2.653	0.154	Yes	Yes	20.49
pVanCC	pVan-Tac	RFP	-2.447	-2.527	-2.373	0.183	Yes	Yes	20.49
pVanCC	pVan-Ttg	RFP	-0.597	-0.676	-0.523	0.661	Yes	Yes	20.49
pTac	pTac-Van	GFP	-2.992	-3.169	-2.801	0.126	No	No	
pTac	pTac-Van	RFP	0.194	-0.234	0.868	1.144	Yes	Yes	20.49

Supplementary Table S12 | Template-promoter YES-gate activation ratios and AND-promoter conditional activation ratios. a, Template and AND-promoter pair identity and concentration matching. **b,** Template-promoter YES-gate activation ratios. **c,** AND-promoter conditional activation ratios. **d,** Ratio-of-ratios, lower-bound flags and response-floor values. R_YES = template [1]/[0] and R_AND = AND [11]/[01]. The delta log2 metric is log2(R_AND) - log2(R_YES). Lower-bound rows use the empirical response floor when the denominator was non-positive.

a

Promoter	Reporter	Channel	State	Mean	n	Alt reporter	Alt mean	Alt n	Alt/formal	Match
pBet-Tac	GFP	GFP	[00]	5973	3	RFP	6472	3	1.083	ind.
pBet-Tac	GFP	GFP	[10]	6075	3	RFP	6858	3	1.129	ind.
pBet-Tac	GFP	GFP	[01]	6206	3	RFP	6398	3	1.031	ind.
pBet-Tac	GFP	GFP	[11]	46516	3	RFP	5710	3	0.123	ind.
pBet-Tac	RFP	RFP	[00]	0.000	3	GFP	0.000	3		ind.
pBet-Tac	RFP	RFP	[10]	0.000	3	GFP	0.000	3		ind.
pBet-Tac	RFP	RFP	[01]	0.000	3	GFP	0.000	3		ind.
pBet-Tac	RFP	RFP	[11]	6139	3	GFP	0.000	3	0.000	ind.
pBet-Van	GFP	GFP	[00]	5876	3	RFP	5876	3	1.000	same
pBet-Van	GFP	GFP	[10]	6180	3	RFP	6180	3	1.000	same
pBet-Van	GFP	GFP	[01]	5741	3	RFP	5741	3	1.000	same
pBet-Van	GFP	GFP	[11]	59190	3	RFP	59190	3	1.000	same
pBet-Van	RFP	RFP	[00]	0.000	3	GFP	0.000	3		same
pBet-Van	RFP	RFP	[10]	0.000	3	GFP	0.000	3		same
pBet-Van	RFP	RFP	[01]	0.000	3	GFP	0.000	3		same
pBet-Van	RFP	RFP	[11]	0.000	3	GFP	0.000	3		same
pPhlF-Tac	GFP	GFP	[00]	6200	3	RFP	5807	3	0.937	ind.
pPhlF-Tac	GFP	GFP	[10]	6986	3	RFP	5361	3	0.767	ind.
pPhlF-Tac	GFP	GFP	[01]	61486	3	RFP	5746	3	0.093	ind.
pPhlF-Tac	GFP	GFP	[11]	71953	3	RFP	7336	3	0.102	ind.
pPhlF-Tac	RFP	RFP	[00]	0.000	3	GFP	0.000	3		ind.
pPhlF-Tac	RFP	RFP	[10]	0.000	3	GFP	0.000	3		ind.
pPhlF-Tac	RFP	RFP	[01]	2988	3	GFP	0.000	3	0.000	ind.
pPhlF-Tac	RFP	RFP	[11]	13564	3	GFP	0.000	3	0.000	ind.
pPhlF-Tet	RFP	RFP	[00]	0.000	3	not available		0		n.a.
pPhlF-Tet	RFP	RFP	[10]	0.000	3	not available		0		n.a.
pPhlF-Tet	RFP	RFP	[01]	16800	3	not available		0		n.a.
pPhlF-Tet	RFP	RFP	[11]	63107	3	not available		0		n.a.
pTac-Tet	GFP	GFP	[00]	7339	3	not available		0		n.a.
pTac-Tet	GFP	GFP	[10]	8818	3	not available		0		n.a.

pTac-Tet	GFP	GFP	[01]	38428	3	not available		0		n.a.
pTac-Tet	GFP	GFP	[11]	74064	3	not available		0		n.a.
pTet-Bet	GFP	GFP	[00]	7753	2	RFP	5253	3	0.678	ind.
pTet-Bet	GFP	GFP	[10]	21701	1	RFP	5957	3	0.274	ind.
pTet-Bet	GFP	GFP	[01]	6455	2	RFP	6100	3	0.945	ind.
pTet-Bet	GFP	GFP	[11]	26924	2	RFP	6583	3	0.245	ind.
pTet-Bet	RFP	RFP	[00]	0.000	3	GFP	0.000	2		ind.
pTet-Bet	RFP	RFP	[10]	0.000	3	GFP	1063	1		ind.
pTet-Bet	RFP	RFP	[01]	0.000	3	GFP	0.000	2		ind.
pTet-Bet	RFP	RFP	[11]	8479	3	GFP	2188	2	0.258	ind.
pTet-PhlF	RFP	RFP	[00]	0.000	3	not available		0		n.a.
pTet-PhlF	RFP	RFP	[10]	3517	3	not available		0		n.a.
pTet-PhlF	RFP	RFP	[01]	0.000	3	not available		0		n.a.
pTet-PhlF	RFP	RFP	[11]	38690	3	not available		0		n.a.
pTet-Ttg	GFP	GFP	[00]	6649	4	RFP	6649	4	1.000	same
pTet-Ttg	GFP	GFP	[10]	12377	2	RFP	12377	2	1.000	same
pTet-Ttg	GFP	GFP	[01]	5806	3	RFP	5806	3	1.000	same
pTet-Ttg	GFP	GFP	[11]	7813	6	RFP	7813	6	1.000	same
pTet-Ttg	RFP	RFP	[00]	0.000	4	GFP	0.000	4		same
pTet-Ttg	RFP	RFP	[10]	0.000	2	GFP	0.000	2		same
pTet-Ttg	RFP	RFP	[01]	0.000	3	GFP	0.000	3		same
pTet-Ttg	RFP	RFP	[11]	1740	6	GFP	1740	6	1.000	same
pTet-Van	GFP	GFP	[00]	7451	3	RFP	7451	3	1.000	same
pTet-Van	GFP	GFP	[10]	8425	3	RFP	8425	3	1.000	same
pTet-Van	GFP	GFP	[01]	6395	3	RFP	6395	3	1.000	same
pTet-Van	GFP	GFP	[11]	8216	3	RFP	8216	3	1.000	same
pTet-Van	RFP	RFP	[00]	0.000	3	GFP	0.000	3		same
pTet-Van	RFP	RFP	[10]	0.000	3	GFP	0.000	3		same
pTet-Van	RFP	RFP	[01]	0.000	3	GFP	0.000	3		same
pTet-Van	RFP	RFP	[11]	28030	3	GFP	28030	3	1.000	same
pTtg-Tac	GFP	GFP	[00]	5386	3	RFP	6858	3	1.273	ind.
pTtg-Tac	GFP	GFP	[10]	5547	3	RFP	6899	3	1.244	ind.
pTtg-Tac	GFP	GFP	[01]	6389	3	RFP	6304	3	0.987	ind.
pTtg-Tac	GFP	GFP	[11]	10002	3	RFP	6092	3	0.609	ind.
pTtg-Tac	RFP	RFP	[00]	0.000	3	GFP	0.000	3		ind.

pTtg-Tac	RFP	RFP	[10]	6.831	3	GFP	0.000	3	0.000	ind.
pTtg-Tac	RFP	RFP	[01]	0.000	3	GFP	0.000	3		ind.
pTtg-Tac	RFP	RFP	[11]	740	3	GFP	0.000	3	0.000	ind.
pVan-Tac	RFP	RFP	[00]	0.000	3	not available		0		n.a.
pVan-Tac	RFP	RFP	[10]	0.000	3	not available		0		n.a.
pVan-Tac	RFP	RFP	[01]	0.000	4	not available		0		n.a.
pVan-Tac	RFP	RFP	[11]	10256	1	not available		0		n.a.
pVan-Ttg	RFP	RFP	[00]	0.000	3	not available		0		n.a.
pVan-Ttg	RFP	RFP	[10]	36787	3	not available		0		n.a.
pVan-Ttg	RFP	RFP	[01]	0.000	3	not available		0		n.a.
pVan-Ttg	RFP	RFP	[11]	36976	3	not available		0		n.a.

b

Promoter	Reporter	State	Formal β1	Alt β1	Alt/formal	Margin check	Formal margin	Δ background	Diagnostic margin	Outcome
pBet-Tac	GFP	[00]	5973	6472	1.083	[11] vs [00]	40542	-762	41304	No
pBet-Tac	GFP	[01]	6206	6398	1.031	[11] vs [01]	40310	-688	40998	No
pBet-Tac	GFP	[10]	6075	6858	1.129	[11] vs [10]	40440	-1149	41589	No
pBet-Tac	GFP	[11]	46516	5710	0.123	minimum corrected margin: [11] vs [01]	40310	-688	40998	No
pPhlF-Tac	GFP	[00]	6200	5807	0.937	[11] vs [00]	65753	1530	64223	No
pPhlF-Tac	GFP	[01]	61486	5746	0.093	[11] vs [01]	10467	1591	8876	No
pPhlF-Tac	GFP	[10]	6986	5361	0.767	[11] vs [10]	64966	1976	62991	No
pPhlF-Tac	GFP	[11]	71953	7336	0.102	minimum corrected margin: [11] vs [01]	10467	1591	8876	No
pTet-Bet	GFP	[10]	21701	5957	n.a.	not assessable	n.a.	n.a.	n.a.	Not assessable
pVan-Tac	RFP	[11]	10256	n.a.	n.a.	not assessable	n.a.	n.a.	n.a.	Not assessable

Supplementary Table S13 | Same-channel alternate-reporter background diagnostic and margin checks. a, State-level diagnostic for each formal reporter-specific state mean from Supplementary Table S2. Parenthetical alternate-reporter values are reported only as diagnostics. Match codes indicate an independent selected source file (ind.), the same selected source file (same; not used as an independent background estimate), or no available alternate context (n.a.). b, Exact independent margin checks for positive same-channel alternate-reporter estimates and non-assessable n = 1 cases. The diagnostic margin is the formal [11]-versus-off-state margin minus the same-channel background differential. These diagnostics were not used as normalisation controls, subtraction terms, or formal reclassification criteria.

References

(1) Meyer, A. J.; Segall-Shapiro, T. H.; Glassey, E.; Zhang, J.; Voigt, C. A. Escherichia coli "Marionette" strains with 12 highly optimized small-molecule sensors. *Nature chemical biology* 2019, *15* (2), 196–204, 10.1038/s41589-018-0168-3. DOI: 10.1038/s41589-018-0168-3.
